# SMOC2 promotes aggressive behavior of fibroblast-like synoviocytes in rheumatoid arthritis through transcriptional and post-transcriptional regulating MYO1C

**DOI:** 10.1038/s41419-022-05479-0

**Published:** 2022-12-13

**Authors:** Di Liu, Ruiru Li, Siqi Xu, Maohua Shi, Yu Kuang, Jingnan Wang, Chuyu Shen, Qian Qiu, Liuqin Liang, Youjun Xiao, Hanshi Xu

**Affiliations:** 1 Department of Rheumatology and Immunology, The First Affiliated Hospital, Sun Yat-sen University, Guangzhou, Guangdong China; 2 Department of Rheumatology, The First People’s Hospital of Foshan, Foshan, Guangdong China

**Keywords:** Rheumatoid arthritis, Autoimmunity

## Abstract

Fibroblast-like synoviocytes (FLSs), play a key role in perpetuating synovial inflammation and bone erosion in rheumatoid arthritis (RA), however, the underlying mechanism(s) of RA FLSs activation and aggression remain unclear. Identifying endogenous proteins that selectively target FLSs is urgently needed. Here, we systematically identified that secreted modular calcium-binding protein 2 (SMOC2), was significantly increased in RA FLSs and synovial tissues. SMOC2 knockdown specifically regulated cytoskeleton remodeling and decreased the migration and invasion of RA FLSs. Mechanistically, cytoskeleton-related genes were significantly downregulated in RA FLSs with reduced SMOC2 expression, especially the motor protein myosin1c (MYO1C). SMOC2 controlled MYO1C expression by SRY-related high-mobility group box 4 (SOX4) and AlkB homolog 5 (ALKHB5) mediated-m^6^A modification through transcriptional and post-transcriptional regulation. Furthermore, intra-articular Ad-shRNA-SMOC2 treatment attenuated synovial inflammation as well as bone and cartilage erosion in rats with collagen-induced arthritis (CIA). Our findings suggest that increased SMOC2 expression in FLSs may contribute to synovial aggression and joint destruction in RA. SMOC2 may serve as a potential target against RA.

**Figure:**
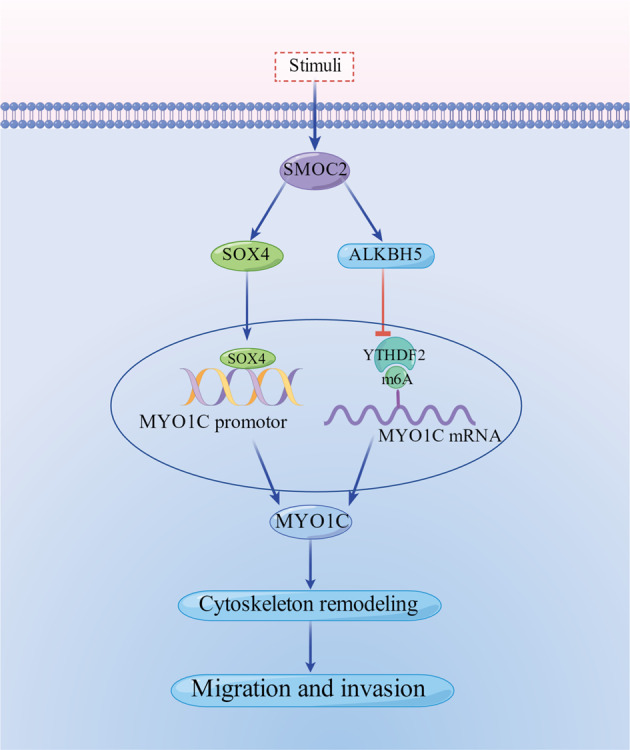
SMOC2-mediated regulation of the synovial migration and invasion in RA FLSs. In RA FLSs, SMOC2 is significantly increased, leading to the increased level of MYO1C via SOX4-mediated transcriptional regulation and ALKBH5-mediated m^6^A modification, thereby causing cytoskeleton remodeling and promoting RA FLSs migration and invasion. The Figure was drawn by Figdraw.

SMOC2-mediated regulation of the synovial migration and invasion in RA FLSs. In RA FLSs, SMOC2 is significantly increased, leading to the increased level of MYO1C via SOX4-mediated transcriptional regulation and ALKBH5-mediated m^6^A modification, thereby causing cytoskeleton remodeling and promoting RA FLSs migration and invasion. The Figure was drawn by Figdraw.

## Introduction

Rheumatoid arthritis (RA) is a chronic autoimmune disease characterized by synovial inflammation and the progressive destruction of joint cartilage and bone [1]. Over the past decades, although multiple synthetic and biological disease-modifying anti-rheumatic drugs (DMARDs) have emerged, a notable percentage of patients have failed to achieve disease remission, especially regarding joint destruction [2]. Thus, new therapeutic targets for RA are urgently needed.

Fibroblast-like synoviocytes (FLSs), which are the most prominent mesenchymal cells in the synovial lining, play a key role in the perpetuation of synovial inflammation and bone erosion [3]. In RA, activated FLSs exhibit a phenotypic transformation into tumor-like cells. These transformed cells display aggressive behavior with enhanced invasiveness and migration ability, increased proliferation and apoptosis resistance. They also produce high levels of inflammatory cytokines, chemokines and matrix metalloproteinases (MMPs), which amplify inflammation and breakdown the extracellular matrix [4, 5]. Increasing evidence suggests that regulating aggressive FLSs may be a promising strategy for controlling joint destruction in RA. However, the underlying mechanism(s) of RA FLS activation and aggression remain unclear; therefore, therapies that identify endogenous proteins which selectively target FLSs have not been available.

Secreted modular calcium-binding protein 2 (SMOC2), which is a member of the secreted protein acidic and rich in cysteine (SPARC) family of matricellular proteins, interacts with matrix proteins, cell surface receptors, cytokines, proteasomes and other effector molecules to regulate cell–cell and cell–microenvironment communication [6]. In recent years, SMOC2 has been reported to be highly expressed in tumor tissues and to promote tumor migration, invasion and growth [7, 8]. In addition to its role in tumor progression, it also regulates wound healing, bone growth, fibrosis and embryogenesis [9–12].

In this study, we identified SMOC2 as one of the most highly upregulated genes in RA FLSs using microarray analysis. We demonstrated that SMOC2 is an important molecule in regulating the migration and invasion of RA FLSs and that myosin1c (MYO1C) may be the target gene of SMOC2. Then, we explored the molecular mechanism by which SMOC2 regulates MYO1C and the possibility of SMOC2 intervention for the treatment of RA in a collagen-induced arthritis (CIA) rat model. We attempt to clarify the underlying mechanisms of SMOC2 and related signaling pathways in synovial invasion and joint destruction in RA, providing a scientific basis for new therapeutic targets in RA.

## Results

### Increased expression of SMOC2 in FLSs and synovial tissues (STs) from patients with RA

**Fig. 1:**
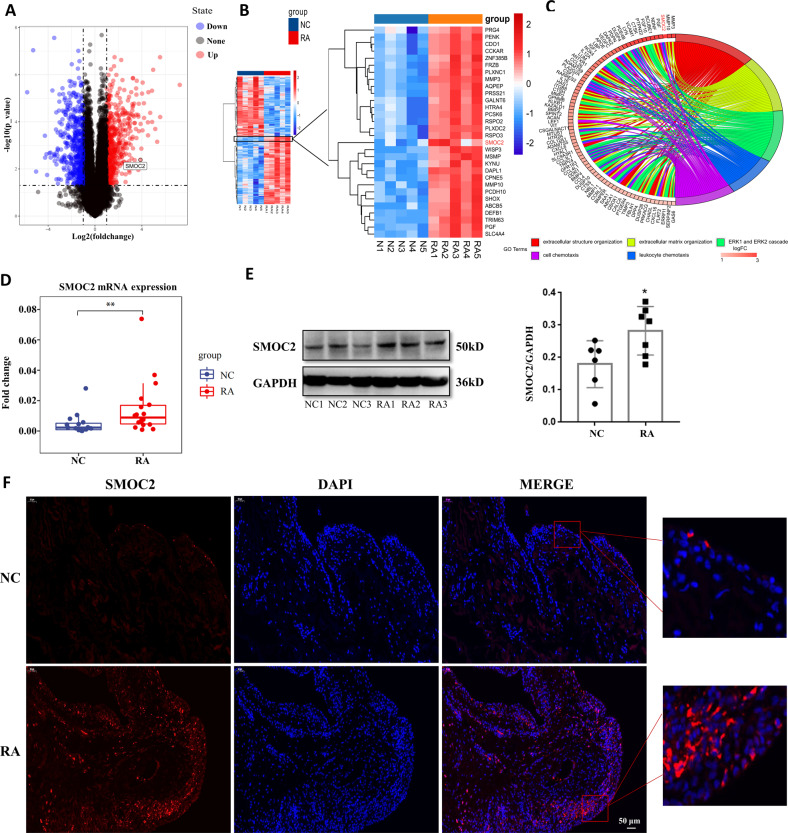
Increased expression of SMOC2 in FLSs and STs from patients with RA. **A** Microarray analysis was performed in FLSs isolated from 5 RA patients and 5 NCs. Volcano plot revealed differentially expressed mRNAs in RA FLSs compared with NCs. **B** Heatmap of significantly altered genes in RA FLSs compared with NCs (fold change-absolute (FC-abs) > 2 and *p* < 0.05). SMOC2 was one of the most upregulated genes in RA FLSs. **C** GO-BP analysis of the upregulated genes between RA FLSs and NC FLSs. **D**, **E** The expression level of SMOC2 was confirmed by RT-qPCR (**D**) and western blot (**E**) in RA FLSs and NC FLSs. **F** Localization and expression of SMOC2 were assessed by immunofluorescence staining in STs from RA patients and NCs. Shown are representative images of SMOC2 (red) and nuclei (blue). Original magnification, ×200. Data are presented as the mean ± SD; **P < 0.01, *P < 0.05.

To reveal the mRNA expression profile of FLSs in RA, we performed microarray analysis of FLSs isolated from 5 RA patients and 5 NC subjects. Volcano plot and heatmap analyses showed that there were 528 upregulated and 476 downregulated genes in RA FLSs compared with NC FLSs (FC-abs > 2 and *p*-value < 0.05) (Fig. 1A, B). In this study, we focused on SMOC2, which was one of the most upregulated genes in RA FLSs. Gene ontology-biological process (GO-BP) analysis of the upregulated genes showed that SMOC2 was significantly enriched in three core GO-BP terms: cell chemotaxis, extracellular structure organization and extracellular matrix organization (Fig. 1C, Supplementary Fig. S1A). We confirmed the increased mRNA and protein expression of SMOC2 in RA FLSs compared with NC FLSs (Fig. 1D, E). We also observed that the protein expression of SMOC2 was increased in RA STs and mainly occurred in the lining layer (Fig. 1F).

### Inhibitory effect of SMOC2 knockdown on RA FLS migration and invasion

**Fig. 2:**
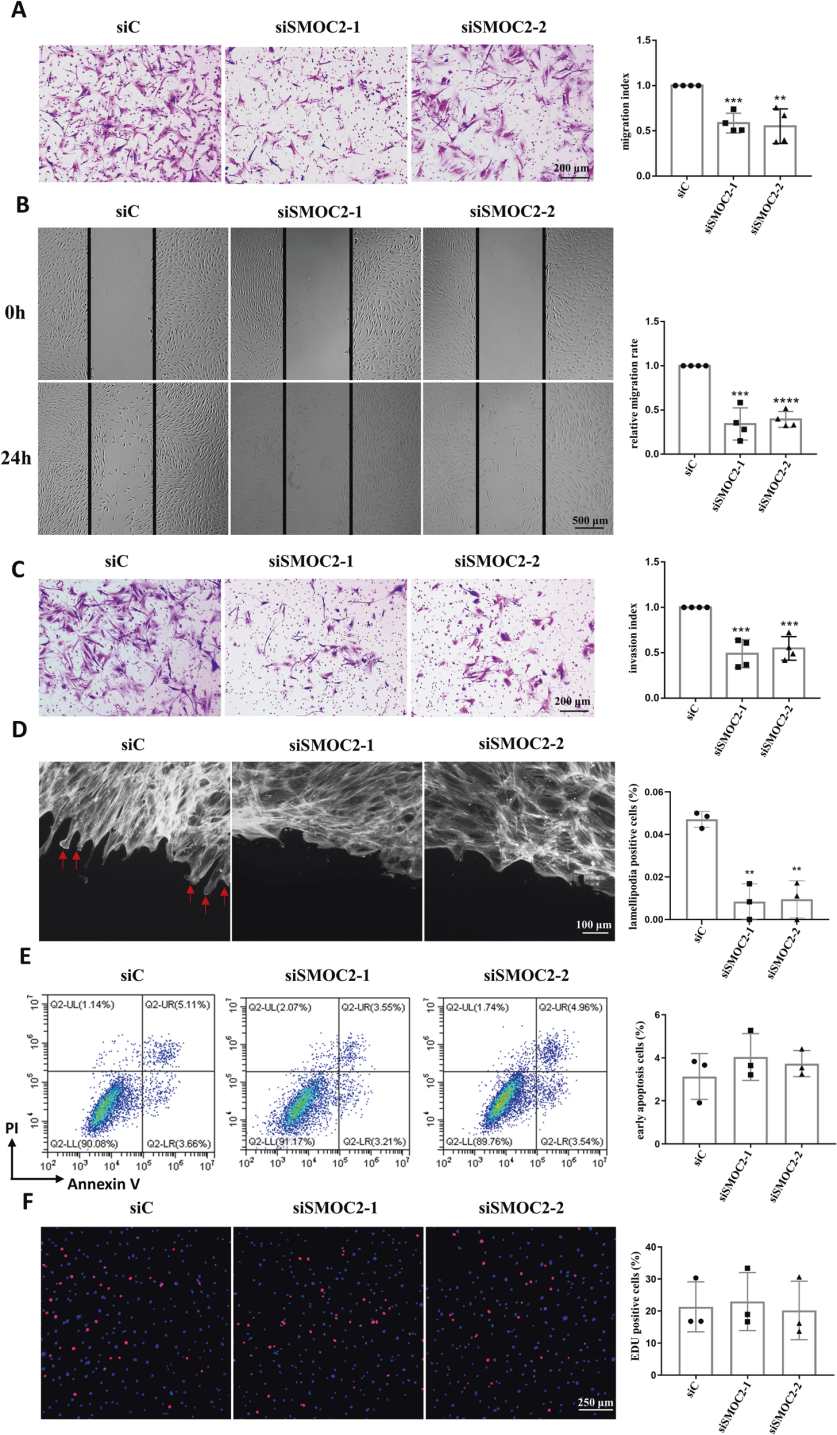
Effects of SMOC2 inhibition on the migration and invasion of RA FLSs. RA FLSs were transfected with SMOC2 siRNA (si-SMOC2-1 or si-SMOC2-2) or control siRNA (siC). **A** Transwell assays were performed to measure the chemotactic migration of RA FLSs. Representative images (original magnification, ×100) are shown. The graphs show the relative migration rates (the number of migrating cells in the siSMOC2 group normalized to that in the siC group) in each group. **B** Wound-healing assays were used to evaluate cell migration. Representative images (original magnification, ×40) are shown. The graphs show the relative migration rates (the number of migrating cells in the siSMOC2 group normalized to that in the siC group). **C** Invasion assays were performed using inserts coated with Matrigel Basement Membrane Matrix. Representative images (original magnification, ×100) are shown. The graphs show the relative invasion rates (relative to that of the negative control group) in each group. **D** Effect of SMOC2 knockdown on lamellipodia formation in RA FLSs. RA FLSs were wounded and incubated in DMEM containing 10% FBS for 6 h and then stained with fluorescent phalloidin to visualize F-actin in migrating FLSs. Representative images (original magnification, ×200) are shown. The arrows indicate lamellipodia formation. The graphs show the lamellipodia-positive rates of RA FLSs. **E** Effect of SMOC2 knockdown on apoptosis in RA FLSs. The cells were stained with annexin V and propidium iodide (PI) and measured by flow cytometry. Representative flow cytometry plots are shown. The graph shows the positive rates of early apoptotic cells. **F** EdU incorporation assays were used to evaluate the effect of SMOC2 knockdown on RA FLS proliferation. Representative images of RA FLSs labeled with EdU (red) and nuclei stained with Hoechst 33342 (blue) are shown. Original magnification, ×100. Data are presented as the mean ± SD of at least 3 independent experiments involving different patients with RA. ****P < 0.0001, ***P < 0.001, **P < 0.01, *P < 0.05.

To explore the function of SMOC2 in RA FLSs, we inhibited SMOC2 expression by using small interfering RNA (siRNA). Among the 3 siRNAs, the inhibitory effects of siRNA-1 and siRNA-2 were more obvious, and these siRNAs were used in the subsequent experiments (Supplementary Fig. S2A, B). Next, we explored whether SMOC2 played an important role in RA FLS migration. We found that SMOC2 knockdown significantly reduced RA FLS migration compared with control siRNA (siC) in a transwell chamber assay (Fig. 2A). A wound healing assay also showed that SMOC2 knockdown resulted in a significant reduction in RA FLS migration compared with control siRNA (Fig. 2B).

Since the invasion of RA FLSs contributes to bone and cartilage destruction in RA, we evaluated the effect of SMOC2 knockdown on RA FLS invasion using Matrigel-coated Transwell membranes. We observed a significant reduction in the invasion capacity of SMOC2-knockdown RA FLSs compared with RA FLSs transfected with control siRNA (Fig. 2C).

The remodeling of the cytoskeleton plays a critical role in cell motility. Thus, we visualized polymerized actin (F-actin) during the migration of RA FLSs in response to wounding by phalloidin staining. We determined that few lamellipodia appeared at the leading edge in FLSs transfected with SMOC2 siRNA but that lamellipodia aggregated at the leading edge in FLSs transfected with control siRNA (Fig. 2D).

Next, we evaluated the role of SMOC2 in the apoptosis and proliferation of RA FLSs. Annexin V-APC/PI double-staining followed by flow cytometry and caspase-3/7 activity determination were used to detect apoptosis in RA FLSs. We demonstrated that SMOC2 knockdown did not result in a significant change in the total rate of early cell apoptosis (Fig. 2E), nor did we observe a change in caspase 3/7 activity (Supplementary Fig. S3A). The EdU staining assay also revealed that SMOC2 knockdown did not affect the proliferation of RA FLSs (Fig. 2F).

Additionally, we evaluated the effect of SMOC2 inhibition on the expression of proinflammatory cytokines, chemokines and MMPs. However, SMOC2 knockdown in RA FLSs failed to affect their expression (Supplementary Fig. S3B).

### MYO1C mediates the role of SMOC2 in regulating RA FLS functions

**Fig. 3:**
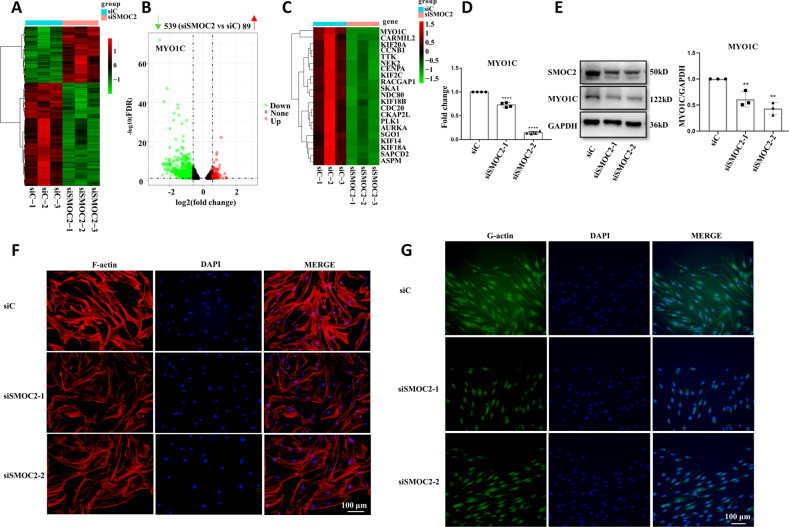
Identification of MYO1C as a downstream target of SMOC2 in RA FLSs. **A**, **B** Heatmap with hierarchical clustering and volcano plot analysis reveal differentially expressed mRNAs between RA FLSs transfected with SMOC2 siRNA (si-SMOC2-2) and those transfected with the control siRNA (siC) (FC-abs >1.5 and *p*-value < 0.05). Among which, MYO1C was the most downregulated gene. **C** Heatmap analysis of cytoskeleton-related genes in SMOC2-knockdown RA FLSs (FC-abs > 1.5 and *p*-value < 0.05). **D** Effect of SMOC2 knockdown on the mRNA expression of MYO1C. **E** Effect of SMOC2 knockdown on the protein expression of MYO1C. **F**, **G** Cell immunofluorescence was performed to detect alterations in the actin cytoskeleton in RA FLSs with SMOC2 knockdown. **F** Phalloidin (red) was used to stain F-actin, while DAPI (blue) was used to stain the nuclei. Original magnification, ×200. **G** DNase I (green) was applied to stain G-actin, while DAPI (blue) was used to stain the nuclei. Original magnification, ×200. Data are presented as the mean ± SD. ****p < 0.0001.

To delineate how SMOC2 regulates the migration and invasion of FLSs in RA, RNA-seq was performed to explore the transcriptome changes in SMOC2-knockdown RA FLSs. We identified significant upregulation of 89 genes and downregulation of 539 genes (FC-abs> 1.5 and *p*-value < 0.05), and MYO1C was the most downregulated gene (Fig. 3A, B). In addition to MYO1C, we also found that some cytoskeleton-related genes were significantly downregulated in the SMOC2-knockdown RA FLSs compared with the control FLSs (Fig. 3C). RT–qPCR and western blot analysis confirmed the decreased mRNA and protein expression of MYO1C in SMOC2-knockdown RA FLSs (Fig. 3D, E). Since MYO1C is a motor protein that directly links cell membranes to the actin cytoskeleton and plays a role in G-actin transport and cytoskeletal rearrangement [13–15], to determine the role of SMOC2 in regulating cytoskeletal change, RA FLSs were stained with Alexa Fluor 568–phalloidin and Alexa Fluor 488–DNase I to visualize F-actin and G-actin. We found that SMOC2 knockdown reduced the amount of F-actin and regulated F-actin cytoskeletal remodeling (Fig. 3F). We also observed a decreased cytoplasm distribution of G-actin in RA FLSs transfected with SMOC2 siRNA compared with the control (Fig. 3G).

**Fig. 4:**
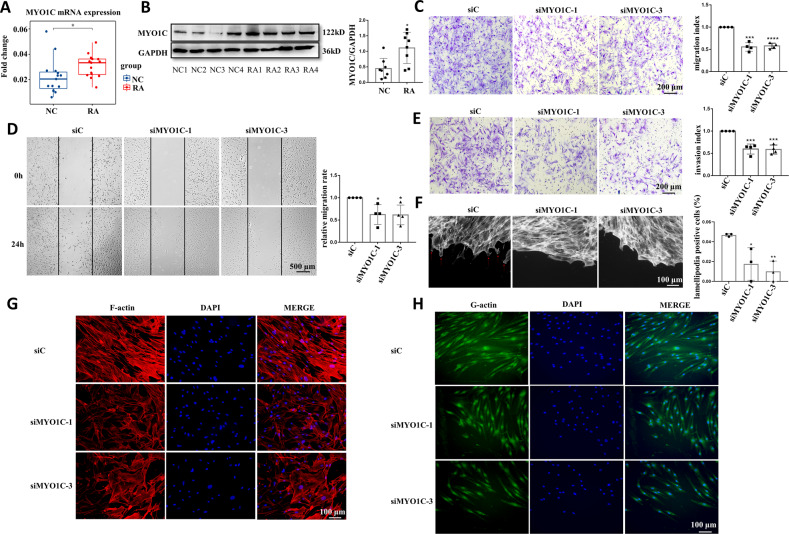
Effect of MYO1C inhibition on migration and invasion of RA FLSs. RA FLSs were transfected with MYO1C siRNA (si-MYO1C-1 or si-MYO1C-3) or the control siRNA (siC). **A** The mRNA expression of MYO1C in RA FLSs and NC FLSs detected by RT-qPCR. **B** The protein expression of MYO1C in RA FLSs and NC FLSs detected by western blot. **C** Transwell assays of RA FLSs were used to measure their chemotactic migration ability. Representative images (original magnification, ×100) are shown. The graphs show the relative migration rates (the number of migrating cells in the siMYO1C group normalized to that in the siC group) in each group. **D** Wound-healing assays were used to evaluate cell migration. Representative images (original magnification, ×40) are shown. The graphs show the relative migration rates (the number of migrating cells in the siMYO1C group normalized to that in the siC group). **E** Invasion assays were performed using inserts coated with Matrigel Basement Membrane Matrix. Representative images (original magnification, ×100) are shown. The graphs show the relative invasion rates (relative to that of the negative control group) in each group. **F** Effect of MYO1C knockdown on lamellipodia formation in RA FLSs. RA FLSs were wounded and incubated in DMEM containing 10% FBS for 6 h and then stained with fluorescent phalloidin to visualize F-actin in migrating FLSs. Representative images (original magnification, ×200) are shown. The arrows indicate lamellipodia formation. The graphs show the lamellipodia-positive rate in RA FLSs. **G**, **H** Effect of MYO1C knockdown on the actin cytoskeleton in RA FLSs. **G** Phalloidin (red) was used to stain F-actin, while DAPI (blue) was used to stain the nuclei. Original magnification, ×200. (**H**) DNase I (green) was applied to stain G-actin, while DAPI (blue) was used to stain the nuclei. Original magnification, ×200. Data are presented as the mean ± SD. ****p < 0.0001, ***P < 0.001, **P < 0.01, *P < *0.05*.

We then determined whether MYO1C is involved in the migration and invasion of RA FLSs. We first observed increased expression of MYO1C in RA FLSs compared with NC FLSs (Fig. 4A, B). Next, we evaluated the role of MYO1C in modulating cytoskeleton alterations and migration in RA FLSs. We constructed 3 different siRNA oligonucleotide sequences to inhibit MYO1C expression. All 3 siRNAs decreased MYO1C mRNA expression, and the inhibitory effects of siRNA-1 and siRNA-3 were more obvious; thus, these siRNAs were used for subsequent experiments (Supplementary Fig. S4A, B). We demonstrated that MYO1C inhibition resulted in significant decreases in RA FLS migration and invasion (Fig. 4C–E). We also observed that few lamellipodia appeared at the leading edge in migrating RA FLSs transfected with MYO1C siRNA in response to wounding but that lamellipodia aggregated at the leading edge in FLSs transfected with siC (Fig. 4F). In addition, the expression of F-actin and G-actin was also decreased along with cytoskeletal remodeling in RA FLSs transfected with MYO1C siRNA (Fig. 4G, H). Collectively, our data suggest that MYO1C mediates SMOC2-induced cytoskeletal remodeling and aggression in RA FLSs.

### SRY-related high-mobility group box 4 (SOX4) mediates the interaction between SMOC2 and MYO1C in RA FLSs

**Fig. 5:**
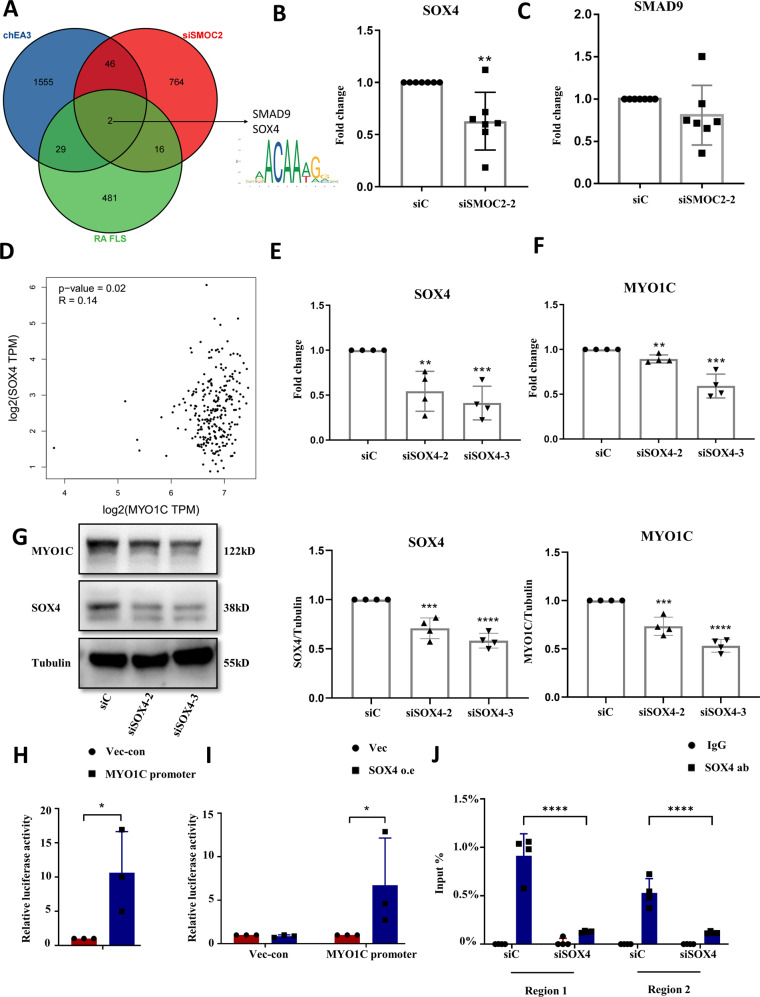
SOX4 mediates the role of SMOC2 in regulating MYO1C transcription in RA FLSs. **A** Venn diagrams show the shared genes between the gene sets of transcription factors of MYO1C, the downregulated genes after SMOC2 knockdown in RA FLSs and the upregulated genes in RA FLSs. **B** Effect of SMOC2 knockdown on the mRNA expression of SOX4. **C** Effect of SMOC2 knockdown on the mRNA expression of SMAD9. **D** Correlation analysis of the mRNA levels of SOX4 and MYO1C in transformed fibroblasts. **E** RA FLSs were transfected with siRNA targeting SOX4 (siSOX4-2, siSOX4-3) or control siRNA (siC) for 48 h and SOX4 mRNA expression was measured by RT-qPCR. **F**, **G** Effect of SOX4 knockdown on the mRNA (**F**) and protein (**G**) expression of MYO1C. **H** The sequence of the MYO1C promotor was inserted into the multiple cloning site (MCS) of a GV238 vector. The relative luciferase activity was calculated as the firefly luciferase activity value/Renilla luciferase activity value. The luciferase reporter plasmids driven by the MYO1C promoter or the negative control plasmids were transfected into 293 T cells. Luciferase reporter assay was performed to analyze the transcriptional activity of the MYO1C promoter in 293 T cells. **I** SOX4 promotes the transcriptional activity of MYO1C in 293 T cells. The luciferase reporter plasmids driven by the MYO1C promoter were transfected into 239 T cells together with a plasmid expressing SOX4 or a negative control plasmid. **J** ChIP-PCR assays were performed to detect the levels of SOX4 binding at the promoter of MYO1C (region 1# (nucleotides −1114 to −1105) and region 2# (nucleotides +1416 to +1425)) in SOX4 knockdown or control RA FLSs. Data are presented as the mean ± SD. ****p < *0.0001*, ***P < 0.001, **P < 0.01, *P < 0.05.

Transcriptional regulation is a core process in regulating gene expression in eukaryotic cells [16]. To explore how SMOC2 modulates MYO1C expression, we predicted MYO1C transcription factors by ChIP-X Enrichment Analysis 3 (CheA3). Figure 5A shows a Venn diagram of the overlaps between MYO1C transcription factors, genes downregulated after SMOC2 knockdown in RA FLSs and genes upregulated in RA FLSs compared with NC FLSs. We found that the transcription factors SOX4 and SMAD family member 9 (SMAD9) were shared between the three gene sets. We further validated that SMOC2 knockdown significantly decreased SOX4 levels but did not affect SMAD9 expression in RA FLSs (Fig. 5B, C). Gene Expression Profiling Interactive Analysis (GEPIA) database analysis revealed that the expression level of SOX4 was positively correlated with MYO1C in transformed fibroblasts (Fig. 5D).

To investigate whether SOX4 regulates MYO1C expression, we designed siRNAs associated with different types of SOX4 target sequences (Fig. 5E). We determined that SOX4 knockdown resulted in a significant decrease in MYO1C mRNA and protein expression (Fig. 5F, G). Next, we generated a MYO1C promoter DNA sequence and cloned it into luciferase reporter plasmids. The 293 T cells transfected with MYO1C promoter elements showed an elevated luciferase signal, which suggested that the dual-luciferase reporter assay worked well (Fig. 5H). Furthermore, we found that SOX4-overexpressing T293 cell lines transfected with the MYO1C promoter had significantly higher luciferase activity than control cells transfected with the MYO1C promoter (Fig. 5I). To further explore whether SOX4 could directly bind the MYO1C promoter, we performed ChIP-PCR assay in SOX4 knockdown or control RA FLSs. As shown in Fig. 5J, SOX4 was associated with the MYO1C promoter from −1114 to −1105 and +1416 to +1425. Furthermore, SOX4 binding to the MYO1C promoter was disrupted in SOX4 knockdown RA FLSs compared with control (Fig. 5J). Taken together, our results suggest that SOX4 can activate the transcriptional activity of the MYO1C promoter and promote MYO1C mRNA expression.

### SMOC2 promotes RA FLS invasiveness via AlkB homolog 5 (ALKHB5)-mediated N6-methyladenosine (m^6^A) modification of MYO1C

**Fig. 6:**
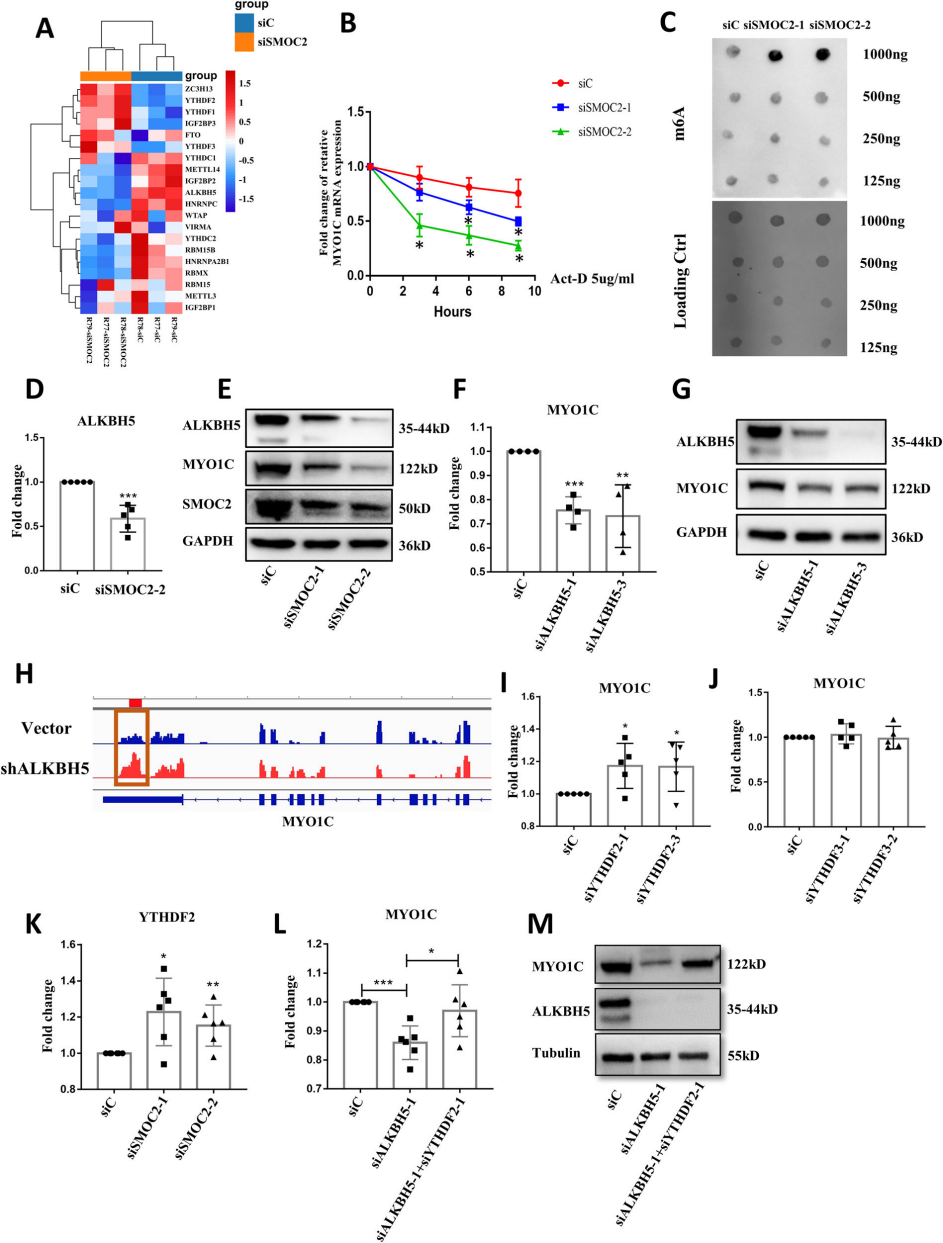
Effect of SMOC2 knockdown on ALKHB5-mediated and YTHDF2-dependent m^6^A modification of MYO1C mRNA. **A** Heatmap analysis of m^6^A-related genes in SMOC2-knockdown RA FLSs (FC-abs >1.5 and *p*-value < 0.05). **B** SMOC2-knockdown RA FLSs were treated with actinomycin D (5 µg/ml) and harvested at 0, 3, 6 and 9 h. The RNA decay rate was used to calculate the stability of MYO1C (normalized to the expression level of MYO1C at 0 h). **C** Effect of SMOC2 knockdown on the global mRNA m^6^A levels in RA FLSs by a RNA m6A dot-blotting assay. **D** Effect of SMOC2 knockdown on the mRNA expression of ALKBH5. **E** Effect of SMOC2 knockdown on the protein expression of ALKBH5, MYO1C and SMOC2. **F** Effect of ALKBH5 knockdown on the mRNA expression of MYO1C. **G** Effect of ALKBH5 knockdown on the protein expression of MYO1C. **H** M^6^A abundance in MYO1C mRNA in ALKBH5 knockdown (colored in red) or the negative control (colored in blue) RA FLSs was detected by MeRIP-seq and visualized by IGV. **I** Effect of YTHDF2 knockdown on the mRNA expression of MYO1C. **J** Effect of YTHDF3 knockdown on the mRNA expression of MYO1C. **K** Effect of SMOC2 knockdown on the mRNA expression of YTHDF2. **L**, **M** Knockdown of YTHDF2 rescued the expression of MYO1C in ALKBH5-depleted RA FLSs. Data are presented as the mean ± SD. ***P < 0.001, **P < 0.01, *P < 0.05.

Recently, epigenetic changes have been suggested to modulate the aggressive behavior of synovial FLSs in RA [3]. Interestingly, we identified multiple m^6^A modification-related genes that were differentially expressed in SMOC2-knockdown RA FLSs compared with control FLSs by RNA-seq (Fig. 6A). We confirmed the increased mRNA expression of fat mass and obesity-associated protein (FTO), the decreased mRNA expression of insulin-like growth factor 2 mRNA-binding protein 2 (IGF2BP2) and methyltransferase-like 14 (METTL14) in SMOC2-knockdown RA FLSs compared with RA FLSs transfected with control siRNA (Supplementary Fig. S5A–C). Next, we investigated whether SMOC2 altered MYO1C mRNA stability in RA FLSs. As shown in Fig. 6B, after actinomycin D treatment, obvious decay of MYO1C mRNA was observed in RA FLSs transfected with SMOC2 siRNA compared with control siRNA, suggesting that SMOC2 also regulates MYO1C by a posttranscriptional pathway. We further validated whether m^6^A modification was involved in the regulation of MYO1C by SMOC2. The m^6^A levels were increased in SMOC2-knockdown RA FLSs (Fig. 6C). Interestingly, among the dysregulated m^6^A-related genes in the heatmap, only the decrease in ALKBH5 resulted in an increase in m^6^A levels (Fig. 6A). We confirmed that SMOC2 knockdown reduced ALKBH5 expression (Fig. 6D, E, Supplementary Figure S6A, B). We further found that ALKBH5 silencing significantly downregulated MYO1C expression (Fig. 6F, G, Supplementary Fig. S6C). To validate the role of ALKBH5 in modulating the m^6^A modification of MYO1C, we performed MeRIP-seq in ALKBH5-knockdown RA FLSs compared with RA FLSs transfected with the vector control and observed a hyper-m6A-peaks for MYO1C upon ALKBH5 silencing (Fig. 6H).

As m^6^A “readers” play an important role related to m^6^A-modified transcripts, we explored the potential reader proteins that regulate MYO1C expression. The RMVar (http://rmvar.renlab.org) online database predicted that YTH domain family 2 (YTHDF2) and YTH domain family 3 (YTHDF3) may bind to MYO1C and regulate its m^6^A modification. We silenced YTDHF2 and YTDHF3 in RA FLSs and found that YTHDF2 knockdown but not YTHDF3 knockdown inhibited MYO1C mRNA expression (Fig. 6I, J, Supplementary Fig. S6D, E). We analyzed the correlation of YTHDF2 or YTHDF3 and MYO1C mRNA expression in RA FLSs. The results showed that YTHDF2 negatively correlated with MYO1C, while the correlation of YTHDF3 with MYO1C was not obvious (Supplementary Fig. S6F, G). We further confirmed that YTHDF2 could bind to the region of m6A peak in MYO1C by using the FIMO software in meme-suite in the Supplementary Fig. S6H. SMOC2 knockdown also increased YTHDF2 expression (Fig. 6K). Next, we performed rescue experiments. ALKBH5 and YTHDF2 were silenced by siRNA in RA FLSs, and YTHDF2 knockdown reversed the ALKBH5 knockdown-induced decrease in MYO1C expression (Fig. 6L, M). Taken together, our results suggest that MYO1C is posttranscriptionally regulated by ALKBH5-mediated m^6^A modification through a YTHDF2-dependent pathway upon SMOC2 silencing in RA FLSs.

### Intraarticular administration of SMOC2 shRNA attenuates the severity of arthritis in rats with CIA

**Fig. 7:**
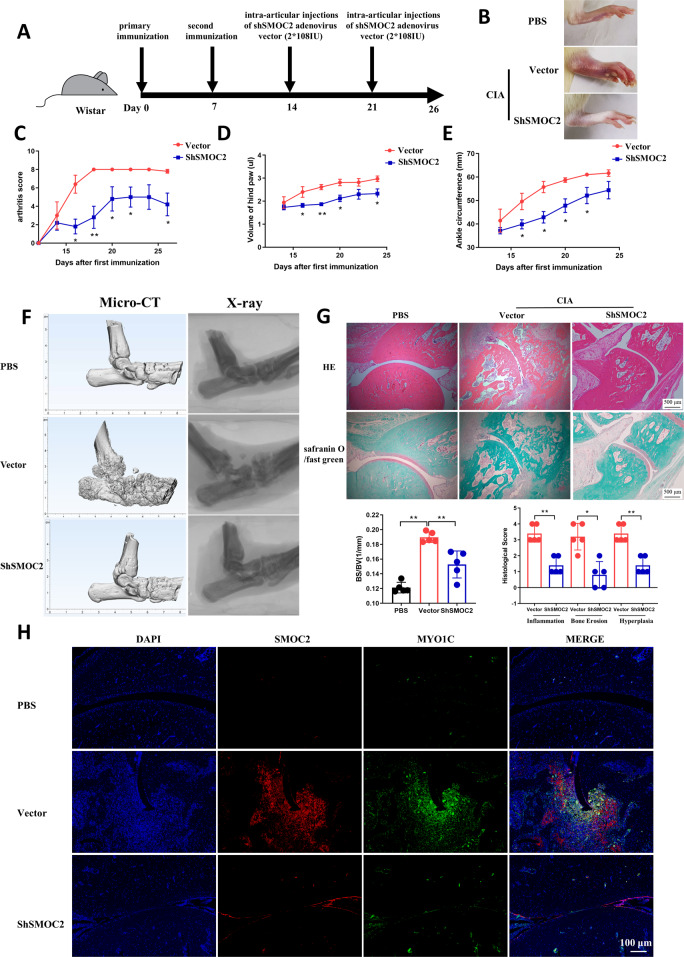
Effect of SMOC2 knockdown on arthritis severity in rats with CIA. **A** Wistar rats were immunized with bovine type II collagen (CII) emulsified in Freund’s adjuvant on Days 0 and Day 7. The CIA rats were injected intra-articularly with 2 * 10^8^ PFU of Ad-shRNA-SMOC2 (*n* = 5) or the control shRNA vector (*n* = 5) on Day 14 and Day 21 in each ankle. **B** Representative images of the CIA rat ankles receiving Ad-shRNA-SMOC2 or the control shRNA vector and the control rat ankles receiving PBS on Day 26. **C**–**E** Effect of SMOC2 knockdown on arthritis scores (**C**), hind paw volumes (**D**) and ankle circumference (**E**) in CIA rats. **F** Micro-CT was performed to evaluate bone erosions in each group. The representative images are shown. The graphs show the quantification of bone erosion by bone surface (BS) divided by bone volume (BV). **G** Hematoxylin and eosin (H&E) and Safranin O (SO)-fast green staining were used for quantification of synovial inflammation, bone erosion and cartilage depletion of CIA rats. Representative images (original magnification, ×100) are shown. **H** Expression of SMOC2 and MYO1C was measured by immunofluorescence staining in synovial tissue from CIA rats. The representative images and quantification of the percentage of SMOC2-positive (red) and MYO1C-positive (green) cells. Original magnification, ×100. Data are presented as the mean ± SD. **P < 0.01, *P < 0.05.

The therapeutic effects of SMOC2 on bone destruction and joint inflammation in vivo were evaluated using rats with CIA. Wistar rats were immunized with bovine type II collagen on Day 0 and Day 7 and intraarticularly injected with 2 х 10^8^ PFU of Ad-shRNA-SMOC2 or the control shRNA vector on Day 14 and Day 21. The arthritis scores, hind paw volumes and ankle circumferences were monitored every 2 days (Fig. 7A). Ad-shRNA-SMOC2 treatment caused significant reductions in paw swelling, arthritis scores, hind paw volumes and ankle circumferences in CIA rats compared with those of CIA rats treated with the control shRNA vector (Fig. 7B–E). SMOC2 inhibition also attenuated joint inflammation, synovial hyperplasia and bone and cartilage damage, as assessed by hematoxylin–eosin (H&E) and Safranin O (SO)-fast green staining and microcomputed tomography (micro-CT) (Fig. 7F, G). We further validated the reduction of SMOC2, MYO1C, ALKBH5 and SOX4 expression in the synovium of rats with CIA with SMOC2 silencing (Fig. 7H, Supplementary Fig. S7A, B). Collectively, our data suggest that SMOC2 inhibition may provide a novel potential target for RA treatment.

## Discussion

In this study, we showed that SMOC2 is a unique endogenous protein highly expressed in RA FLSs and RA STs. Importantly, SMOC2 knockdown regulated cytoskeletal remodeling and decreased aggressive behavior in RA FLSs. Mechanistically, we identified two independent modes by which SMOC2 controls MYO1C expression. One is SOX4-mediated transcriptional regulation, and the other is ALKBH5-mediated m^6^A modification at the posttranscriptional level. Interestingly, intraarticular Ad-shRNA-SMOC2 treatment attenuated synovial inflammation and bone and cartilage erosion in rats with CIA. These findings suggest that SMOC2 may serve as a potential target against RA.

SMOC2, which is a member of the SPARC family of matricellular proteins, is involved in the progression of multiple cancers. For instance, increased SMOC2 is associated with lymph node metastasis, distant metastasis, decreased chemosensitivity and a poor prognosis in endometrial cancer [8, 17]. SMOC2 promotes proliferation and cell cycle progression in hepatocellular carcinoma cells [18]. In lung adenocarcinoma, SMOC2 functions as a secreted prometastatic factor [19]. SMOC2 is also considered an intestinal stem cell signature gene and plays a key role in colorectal cancer cell motility, proliferation and liver metastasis [7]. In our study, we detected high SMOC2 expression in RA FLSs, and SMOC2 knockdown inhibited the migration and invasion of RA FLSs. SMOC2 knockdown also decreased the severity of arthritis in CIA rats. These findings suggest that increased SMOC2 contributes to the aggressive phenotype in RA FLSs.

To explore how SMOC2 modulates the aggressive behaviors of RA FLSs, we performed RNA-seq to search for downstream targets. We identified MYO1C as a downstream target protein of SMOC2 in RA FLSs. MYO1C is an unconventional F-actin-binding motor protein, and its head motor region interacts with G-actin. The tail region connects to the cell membrane, thereby driving actin transport and affecting cytoskeleton remodeling. Emerging evidence indicates that MYO1C participates in various cellular biological processes by affecting the dynamics of the actin cytoskeleton, such as autophagosome–lysosome fusion, glucose transporter 4 vesicle transport, antigen presentation and cell motility [14, 20–22]. In endothelial cells, MYO1C delivers G-actin to the leading edge of the cell and ultimately affects cell migration [15]. In addition to direct regulation, MYO1C also indirectly regulates actin cytoskeleton dynamics by transporting specific signaling pathway molecules, such as mammalian target of rapamycin (mTOR) and NF-κB essential regulatory protein, to cell membranes [23, 24]. These studies indicate a critical role of MYO1C in regulating actin cytoskeleton remodeling and cell motility. Herein, we demonstrated that the expression of MYO1C was increased in RA FLSs compared with NC FLSs and that MY1OC knockdown decreased the migration and invasion of RA FLSs. We also found that MYO1C knockdown reduced the formation of lamellipodia and the distribution of G-actin and F-actin in RA FLSs. We further demonstrated that SMOC2 knockdown decreased the formation of lamellipodia and the distribution of G-actin and F-actin in RA FLSs. Collectively, our findings suggest that MYO1C mediates SMOC2-induced cytoskeletal remodeling and the aggressive phenotype in RA FLSs.

We next explored the underlying mechanisms by which SMOC2 controls MYO1C expression. Since our data showed that SMOC2 knockdown reduced MYO1C mRNA expression, we first explored whether SMOC2 modulated MYO1C gene transcription. We found that the transcription factor SOX4 could activate the transcriptional activity of the MYO1C promoter and positively regulate MYO1C mRNA expression, and SMOC2 knockdown decreased SOX4 expression, suggesting that SMOC2 controls MYO1C expression through SOX4-mediated gene transcription. Consistent with our findings, previous studies have shown that SOX4 is a critical transcription factor involved in the progression of multiple cancers, and it has also been found to be upregulated in mouse arthritis models and CD4 + T cells in the inflamed joints of RA patients [25–28]. Moreover, in transgenic mice expressing human TNFα, SOX4/11 amplifies TNFα-induced FLS aggressiveness [29].

Additionally, we determined whether SMOC2 modulates MYO1C expression in a posttranscriptional way. Since increasing evidence indicates an important role of RNA modification in regulating posttranscriptional control, we investigated whether m^6^A, as the most abundant type of internal mRNA modification, is involved in the modulation of SMOC2-induced MYO1C expression. First, we found that SMOC2 knockdown increased the m^6^A level and affected MYO1C mRNA stability in RA FLSs. Second, we demonstrated that the expression of ALKBH5, which is a key m^6^A demethylase, was decreased in SMOC2-knockdown RA FLSs and that ALKBH5 silencing resulted in elevated m^6^A levels and decreased MY1OC expression. Third, we determined that knockdown of YTHDF2, which is a m^6^A reader that promotes the degradation of transcripts by binding to m^6^A-containing mRNA, rescued the expression of MYO1C in ALKBH5-knockdown RA FLSs [30]. Collectively, our data suggest that SMOC2-induced MYO1C expression could be posttranscriptionally regulated by ALKBH5-mediated m^6^A modification in a YTHDF2-dependent manner in RA FLSs. Indeed, ALKBH5 has been shown to regulate transcriptional modification, maintain mRNA stability, and ultimately promote tumor progression in numerous tumors [31–33].

In summary, we identified SMOC2 as the key molecule that controls aggressive behavior in RA FLSs. We explored whether MYO1C mediates the role of SMOC2 in regulating RA FLS functions. Furthermore, we demonstrated that SMOC2 modulates MYO1C expression at both the SOX4-mediated transcriptional level and the ALKBH5-mediated m^6^A modification level. Our findings suggest that increased SMOC2 expression in FLSs may contribute to synovial aggression and joint destruction in RA.

## Method

### Preparation of human STs and FLSs

STs were obtained from 30 active RA patients (25 women and 5 men, aged 57.3 ± 9.5 years) who underwent synovectomy of the knee joint at The First Affiliated Hospital, Sun Yat-sen University. All RA patients met the 2010 revised RA classification criteria of the American College of Rheumatology/European League Against Rheumatism [34]. We obtained normal control (NC) STs from 20 age- and sex-matched participants who underwent traumatic single above-knee amputation and had no history of acute or chronic arthritis. This study was approved by the ethics committee at First Affiliated Hospital of Sun yat-sen university (Guangzhou, Guangdong, China). All participants signed an informed consent form, and the detailed information of all patients was listed in Supplementary Table 1.

STs were cut into small pieces and placed in a cell-culture dish for 4–6 h of adhesion. The cells were cultured in Dulbecco’s modified Eagle’s medium (DMEM/F12) containing 10% fetal bovine serum (FBS) at 5% CO_2_ and 37 °C. FLSs from passages 4–6, which were a homogeneous cell population, were used in our experiments.

### Microarray analysis

Microarray analysis was performed on NC FLSs (*n* = 5) and RA FLSs (*n* = 5). Arraystar Human LncRNA Microarray V3.0 was used for the global profiling of gene expression. RNA labeling, array hybridization RNA isolation and microarray processing were performed as previously described [35]. After quantile normalization of the raw data, mRNAs with a *P* value <0.05 and FC-abs >1.5 between the two groups (RA vs. NC) were regarded as differentially expressed genes. Volcano plot and cluster heatmap analyses were performed with the R package ggplot2. GO enrichment analyses were performed with the R package clusterProfiler. The microarray data have been deposited into the National Center for Biotechnology Information (NCBI) Gene Expression Omnibus (GEO) database (GEO GSE181614).

### RNA isolation and quantitative RT–qPCR

Total RNA was extracted with the Takara PrimeScript RT Reagent Kit (Takara Bio, Japan) according to the manufacturer’s instructions, and RT–qPCR was carried out with LC480 cells (Roche, USA). All primers used in our study are listed in Supplementary Table 2. Target gene expression relative to glyceraldehyde-3-phosphate dehydrogenase (GAPDH) expression was calculated by the 2^−△△CT^ method.

### Western blot analysis

FLSs were lysed with RIPA buffer containing protease inhibitors and phosphatase inhibitors, and the protein concentrations were detected with a BCA protein assay kit (P0011, Beyotime, China). Equal amounts of protein were loaded onto 10% polyacrylamide gels and transferred onto polyvinylidene difluoride (PVDF) membranes, and the membranes were blocked with TBS/Tween-20 containing 5% nonfat milk for 1 h. Then, the membranes were incubated with the primary antibodies anti-SMOC2 (ab56088, Abcam, 1:1000, USA), anti-MYO1C (ab194828, Abcam, 1:1000), anti-ALKBH5 (ab195377, Abcam, 1:1000) and anti-GAPDH (G8795, Millipore Sigma, 1:3000, USA) in antibody diluent at 4 °C overnight. The membranes were incubated with the appropriate secondary antibodies for 1 h at room temperature (RT), and the bands were visualized using ECL (Bio–Rad, USA). Signals from the bands were quantified by ImageJ software (National Institutes of Health, USA).

### Transfection of siRNAs

The SMOC2, MYO1C, SOX4, ALKBH5, YTHDF2 and YTHDF3 siRNAs and control siRNAs were obtained from RiboBio. The target sequences of each siRNA are listed in Supplementary Table 3. FLSs at 60–70% confluence were transiently transfected with the above siRNAs (100 nM) or the corresponding NC using Lipofectamine 3000 (Thermo Fisher Scientific, USA) according to the manufacturer’s protocol. Experiments were performed 48–72 h after transfection.

### Detection of cell migration and invasion

The migration assay was performed using the Boyden chamber method with a 6.5 mm-diameter and 8.0 μm-pore size filter (Transwell; Corning Labware Products, USA). Briefly, 0.6 ml DMEM containing 20% FBS was added to the lower chamber as a chemoattractant, and 2 × 10^4^ FLSs suspended in 300 μL serum-free DMEM were seeded into the upper chamber. After incubation for 8 h at 37 °C under 5% CO_2_, the filters were fixed in methanol for 15 min and stained with 0.1% crystal violet (Sigma–Aldrich, USA) for 20 min. For invasion assays, similar experiments were conducted using inserts coated with Matrigel (BD Biosciences, USA), and 4 × 10^4^ FLSs suspended in 600 µl serum-free DMEM were seeded into the upper chamber. The plate was incubated at 37 °C and 5% CO2 for 16 h. Migrated and invaded cells were quantified by using an optical microscope (Olympus BX53, Japan) and counted as the mean number of cells per 10 random fields in each assay.

### Wounding migration

For the wounding migration assay, FLSs were seeded into 12-well plates (NEST Biotechnology, Wuxi, China) and cultured to 90% confluence. Then, the cells were starved for 12 h, scratched with 200 μL pipette tips and treated with DMEM containing 10% FBS for 24 h. Migration was quantified by counting the numbers of FLSs that had moved beyond a reference line.

### EdU proliferation assays

Cells were seeded into 96-well plates in DMEM complete media and cultured to 80–90% confluence in each well. Then, they were incubated with 50 μM EdU buffer in DMEM complete media at 37 °C for 8 h. The Cell-Light EdU DNA Cell Proliferation Kit (RiboBio, China) was used according to the manufacturer’s instructions to detect the cell proliferation ability.

### Apoptosis assays

Cell apoptosis was detected by staining the cells with an Annexin V-FITC/Propidium Iodide (PI) Apoptosis Detection kit (BD Biosciences, USA). Briefly, FLSs were suspended in PBS at a concentration of 1 × 10^6^ cells/mL. Then, 100 μL of the cell suspension was reacted with 5 μL of Annexin V-FITC and 5 μL of PI for 20 min in darkness at RT. Apoptotic cells were detected by flow cytometry (Beckman Coulter, USA) and analyzed with FlowJo software (FlowJo, USA).

### Caspase 3/7 activity assays

FLSs were plated in 96-well plates, and caspase-3/7 activity was measured by using a caspase-Glo 3/7 Assay (Promega, USA) kit according to the manufacturer’s instructions. Caspase-Glo 3/7 Reagent was added to each well, and the plates were incubated at RT. Finally, the luminescence intensity of each sample was detected by a luminometer.

### Immunofluorescence (IF) staining

For IF analysis, paraffin-embedded synovial tissues were deparaffinized, rehydrated, permeabilized and subjected to antigen retrieval. The synovial tissues were blocked with 5% bovine serum albumin (BSA) in phosphate-buffered saline (PBS) for 1 h at RT and then incubated with primary antibodies against SMOC2 (sc-376104, Santa Cruz, 1:50, CA), MYO1C (ab194828, Abcam, 1:50), SOX4 (ab86809, Abcam, 1:50), and ALKBH5 (ab195377, Abcam, 1:200) at 4 °C overnight. Nuclei were stained with DAPI, and images were obtained by fluorescence microscopy (Olympus BX53, Japan).

For cell IF analysis, cells growing on glass coverslips were fixed with 3.7% formaldehyde at RT for 15 min and then permeated with 0.1% Triton X-100 in PBS for 10 min. For the detection of pseudopodia formation, FLSs were stained with Alexa Fluor 546 rhodamine-phalloidin (Molecular Probes, Thermo Fisher Scientific, USA), and for the measurement of monomeric G-actin, FLSs were stained with Fluorescent DNase I (Invitrogen, CA). Nuclei were stained with DAPI, and images were obtained by fluorescence microscopy (Olympus BX53, Japan).

### RNA sequencing (RNA-seq) and bioinformatics analysis

The detailed processes of RNA isolation, sequencing library preparation, sequencing and bioinformatics analysis were performed as previously described [35]. Briefly, total RNA was isolated from SMOC2-knockdown or control FLSs using TRIzol reagent. RNA library preparation was performed using a KAPA Stranded RNA-Seq Library Prep Kit (Illumina). All samples were sequenced on an Illumina NovaSeq 6000 platform. Sequence reads were mapped to UCSC human genome version hg38 as the reference genome. Differentially expressed gene analysis of the RNA-seq data was performed with the R package DESeq2. Volcano plot and cluster heatmap analyses were performed with the R package ggplot2.

### Luciferase reporter assays

MYO1C promoters containing GV238 plasmids and SOX4 overexpression GV712 plasmids or negative control plasmids (Jikai, China) were transiently cotransfected into 293 T cells with Lipofectamine 3000 (Invitrogen, CA). After 48 h of transfection, firefly and Renilla luciferase activities were detected by the Dual-luciferase Reporter Assay system (Promega, USA). The expression efficiency of each well was calculated as the firefly luciferase activity value/Renilla luciferase activity value.

### Chromatin immunoprecipitation (ChIP) assay

The ChIP assay kit (CST, USA) was used for ChIP assay according to the manufacturer’s instructions. In brief, RA FLSs transfected with siRNA targeting SOX4 (siSOX4-3) or control siRNA were cross-linked with 1% formaldehyde and lysed in the lysis buffer. Then the lysis was sonicated on ice to generate 200 to 500 bp DNA fragments. After centrifugation, the supernatant containing chromatin was immunoprecipitated with anti-SOX4 (ab86809, Abcam) or isotype control IgG antibodies overnight at 4 °C. Immunoprecipitated DNAs were purified and subjected to RT-qPCR analysis. The ChIP primers were listed in Supplementary Table 2.

### Methylated RNA immunoprecipitation sequencing (MeRIP-seq)

Total RNA was isolated from ALKBH5-deleted RA FLSs and RA FLSs transfected with control vectors. RNA fragmentation and m6A immunoprecipitation were performed by using the MeRIP m6A Kit (Merck Millipore, USA) according to the manufacturer’s instructions. The RNA sequencing libraries for input mRNA (RNA-seq) and MeRIP-seq were constructed with the NEBNext Ultra RNA library Prep kit for Illumina (NEB, USA), followed by sequencing on an Illumina NextSeq 500 sequencer. The sequencing reads were aligned to the reference genome (human genome GRCh37/hg19) by HISAT2 software (v2.0.4). m6A peaks were detected by MACS software, and the m6A peak distribution was visualized by Integrative Genomics Viewer (IGV) software.

### RNA m^6^A dot blot assay

mRNA was denatured at 95 °C for 3 min and cooled, and samples (1000 ng, 500 ng, 250 ng, or 125 ng) were then spotted onto an Amersham Hybond-N + membrane (GE Healthcare, USA). The membranes were UV cross-linked and stained with 0.2% methylene blue in 0.3 M sodium acetate (pH 5.2), which was used to ensure equal amounts of input RNA content among the different groups. Then, the membranes were blocked and incubated with a m^6^A antibody (56593, CST, 1:1000, USA) at 4 °C overnight. Subsequently, the membranes were incubated with HRP-conjugated goat anti-rabbit IgG (1:3000, CST, USA). The m^6^A dots on the membrane were visualized by the MiniChemi 610 chemiluminescence system (Sagecreation, China).

### RNA stability

For the RNA stability assay, FLSs were treated with 5 μg/ml actinomycin D (Act-D, Selleck, China) for 0, 3, 6, and 9 h. The FLSs were lysed in TRIzol at each time point. The MYO1C mRNA level was measured by RT–qPCR, and GAPDH was used for normalization.

### Establishment of CIA and intra-articular Ad-shRNA-SMOC2 therapy

To establish the CIA model, Wistar rats (male, 9 weeks) were immunized intradermally at the base of the tail with bovine type II collagen (CII) emulsified in Freund’s adjuvant on Day 0 and Day 7, as previously described [36]. CIA rats were randomly chosen to receive an intraarticular injection of 2*10^8^ PFU of Ad-shRNA-SMOC2 (*n* = 5) or the control shRNA vector (*n* = 5) in each ankle on Day 14 and Day 21. In addition, the control group received intraarticular injections of an equal volume of PBS (*n* = 5). The arthritic scores, hind paw volumes and ankle circumferences were evaluated every other day through Day 26 as previously described. On Day 26, all rats were anesthetized, and their hind limbs were removed and fixed in 4% paraformaldehyde for further histological and radiographic analyses.

### Microcomputed tomography (micro-CT) scanning

Rat ankles were fixed in formalin. After fixation, the samples were scanned by a Skyscan1176 Micro-CT (Bruker, Germany) at 65 kV/380 μA, with a voxel size of 25 μm and a 1 mm aluminum filter. X-ray projections were obtained at a 0.3° shooting pitch with a 180° tomographic rotation and a combination of four average frames. Three-dimensional images of the region of interest were reconstructed by Materialise 3-matic (Materialise, Belgium). The data were analyzed by CT-Analyzer software (Bruker, Germany), and images were generated by CTVOX software (Bruker, Germany). The bone surface (BS)/bone volume (BV) (1/mm) value was used to quantify bone erosion as previously described [37].

### Histological analysis

Rat hind paws were fixed in formalin, decalcified with ethylene diaminetetraacetic acid (EDTA) and embedded in paraffin. The tissues were cut into 4 μm sections and stained with hematoxylin and eosin (H&E) and safranin O-fast green to assess joint inflammation and bone and cartilage damage. The stained tissues were imaged by microscopy (Olympus BX53, Japan). Histological analyses were performed as described previously [38].

### Statistics

Representative data are expressed as the mean ± SD. In vitro experiments were independently repeated at least 3 times. To reduce baseline variability between independent experiments, the quantitative analyses of immunoblots and mRNA expression were normalized. The data were normalized to the FC divided by the mean of the control. The proper post hoc test (for groups of ≥ 3) was applied to compare between the groups for calculating statistical significance. The normality was checked on the raw data. If the data meet the criteria of normal distribution, two-tailed student’s t-test or One-way analysis of variance (ANOVA) were used for two groups and multiple comparisons, respectively. If the data do not meet the criteria of normal distribution, Mann-Whitney test or Kruskal-Wallis test was performed or two groups and multiple comparisons, respectively. A *P*-value < 0.05 was considered significant. Statistical analyses were performed with GraphPad Prism software v 9.0.

## Supplementary information

supplement table

Supplement Figure S1

Supplement Figure S2

Supplement Figure S3

Supplement Figure S4

Supplement Figure S5

Supplement Figure S6

Supplement Figure S7

aj-checklist

Original Data File

## Data Availability

All datasets generated and analysed during this study are included in this published article and its Supplementary Information files. Additional data are available from the corresponding author on reasonable request.
