# Supplementary material for: SMOC2 promotes aggressive behavior of fibroblast-like synoviocytes in rheumatoid arthritis through transcriptional and post-transcriptional regulating MYO1C
Source: Cell Death Dis. 2022 Dec 13;13(12):1035. doi: 10.1038/s41419-022-05479-0 (PMC9747908; doi:10.1038/s41419-022-05479-0)
Supplement: Supplementary file 1 — supplement table [file 41419_2022_5479_MOESM1_ESM.doc]

| Age, yrs (mean±SD) | 57.3±9.5 |
| --- | --- |
| Female, 𝑛 (%) | 25（83.3） |
| Male, n (%) | 5（16.7） |
| Disease duration, yrs (mean±SD) | 9.7±11.0 |
| Rheumatoid factor-positive, 𝑛 (%) | 25（83.3） |
| Anti-CCP-positive, 𝑛 (%) | 22（73.3） |
| DAS28 (CRP) (mean±SD) | 5.4±1.4 |
| Previous medications, 𝑛 (%) | 18（60.0） |
| Prednisone (<10mg/d) | 13（43.3） |
| Methotrexate | 8（26.7） |
| Leflunomide | 4（13.3） |
| Sulfasalazine | 1（3.3） |
| Hydroxychloroquine | 4（13.3） |
| Tofacitinib | 2（6.7） |

**supplementary tables**

**Table S1. Demographic and clinical features of patients with active RA**

**Table S2. Sequences of RT-PCR primers**

| Name | Forward | Reverse |
| --- | --- | --- |
| SMOC2 | GGAGTTTCAGCAAGTGTTCATTC | GCGGCATCATCTGTTTTTCC |
| MYO1C | CTGTGATCTGGTGGAGGAGAAG | GTCAGGAAGTGTGGATGGTGCT |
| SOX4 | GCACTAGGACGTCTGCCTTT | ACACGGCATATTGCACAGGA |
| ALKBH5 | CCAGCTATGCTTCAGATCGCCT | GGTTCTCTTCCTTGTCCATCTCC |
| YTHDF2 | TAGCCAGCTACAAGCACACCAC | CAACCGTTGCTGCAGTCTGTGT |
| YTHDF3 | GCTACTTTCAAGCATACCACCTC | ACAGGACATCTTCATACGGTTATTG |
| SMAD9 | CCCTGCCTTATCATGCCACA | AGGGTCGGTGAACCCATCTA |
| IL1β | ATGATGGCTTATTACAGTGGCAA | GTCGGAGATTCGTAGCTGGA |
| IL6 | ACTCACCTCTTCAGAACGAATTG | CCATCTTTGGAAGGTTCAGGTTG |
| IL8 | ACTGAGAGTGATTGAGAGTGGAC | AACCCTCTGCACCCAGTTTTC |
| CCL2 | CAGCCAGATGCAATCAATGCC | TGGAATCCTGAACCCACTTCT |
| MMP1 | CTCTGGAGTAATGTCACACCTCT | TGTTGGTCCACCTTTCATCTTC |
| MMP2 | AGCGAGTGGATGCCGCCTTTAA | CATTCCAGGCATCTGCGATGAG |
| MMP3 | TGTAAAGAAACCTTCCTGCAA | TTTAAAACACAGTATGCCCAA |
| MMP9 | TGTACCGCTATGGTTACACTCG | GGCAGGGACAGTTGCTTCT |
| MMP13 | TCCTGATGTGGGTGAATACAATG | GCCATCGTGAAGTCTGGTAAAAT |
| FTO | CCAGAACCTGAGGAGAGAATGG | CGATGTCTGTGAGGTCAAACGG |
| IGF2BP2 | GTTGGTGCCATCATCGGAAAGG | TGGATGGTGACAGGCTTCTCTG |
| GAPDH | GCACCGTCAAGGCTGAGAAC | TGGTGAAGACGCCAGTGGA |
| MYO1C promoter region1 | CCACTGGGTGGAAACAGGAA | TCCTCCGGGAAGCACATCTA |
| MYO1C promoter region2 | GACTGGAGGGCTCAGTCTCA | GCGTTTCAACAGTACCCCCA |

**Table S3 Sequences of siRNA**

| SMOC2 | siRNA-1 | GGACATTATTCTCCTGATC |
| --- | --- | --- |
|  | siRNA-2 | CACAGAACCAGCTCAAGTA |
|  | siRNA-3 | TCAAGTACATGCCAATGTT |
| MYO1C | siRNA-1 | GTGGCCACATCCTCAGTTA |
|  | siRNA-2 | GCCAAAGTCTCCTCCATCA |
|  | siRNA-3 | CTCTGACAGTCATTGATTT |
| SOX4 | siRNA-1 | GCTTCCTACCTTGCAACAA |
|  | siRNA-2 | CGAGAAACTTGCATTGGAA |
|  | siRNA-3 | GGTGCAGCAAACCAACAAT |
| YTHDF2 | siRNA-1 | GACCAAGAATGGCATTGCA |
|  | siRNA-3 | GGTAGCGGGTCCATTACTA |
| YTHDF3 | siRNA-1 | CATACATCGTTCCATTAAA |
|  | siRNA-2 | GTCAGTGCTTCACCTTCTA |
| ALKBH5 | siRNA-1 | GATCGCCTGTCAGGAAACA |
|  | siRNA-3 | GCTGCAAGTTCCAGTTCAA |

**supplementary legends**

**Fig. S1 Pathway analyses of the upregulated genes between RA FLSs and normal control (NC) FLSs.** (A) GO-BP analysis of the upregulated genes between RA FLSs and NC FLSs showed that 3 of Top 5 enrichment pathways were significantly enriched with SMOC2 gene.

**Fig. S2 Efficiency of SMOC2 knockdown.** (A) RA FLSs were transfected with SMOC2 siRNA (siSMOC2-1, siSMOC2-2, siSMOC2-3) for 48 hours and then SMOC2 mRNA expression was measured by RT-qPCR analysis. (B) RA FLSs were transfected with SMOC2 siRNA (siSMOC2-1, siSMOC2-2) for 72 hours and then SMOC2 protein expression was measured by western blot. Data shown are the mean ± SD of densitometry quantification (right panel) from 4 independent experiments involving 4 different patients with RA. ****P* < 0.001, *****p* < 0.0001.

**Fig. S3 Effect of SMOC2 knockdown on apoptosis and the expression of proinflammatory cytokines, chemokines and MMPs in RA FLSs.** (A) Caspase-Glo 3/7 Assay was used to detect the caspase 3/7 activity. Data are expressed relative to the values in the siC group. (B) Effect of SMOC2 knockdown on the expression of IL-1β, IL-6, IL-8, CCL2, MMP1, MMP2, MMP3, MMP9, MMP13. Data are presented as the mean ± SD of 4 independent experiments involving 4 different patients with RA.

**Fig. S4 Efficiency of MYO1C knockdown.** (A) RA FLSs were transfected with MYO1C siRNA (siMYO1C-1, siMYO1C-2, siMYO1C-3) for 48 hours and then MYO1C mRNA expression was measured by RT-qPCR analysis. (B) RA FLSs were transfected with MYO1C siRNA (siMYO1C-1, siMYO1C-3) for 72 hours and then MYO1C protein expression was measured by western blot. Data shown are the mean ± SD from at least 3 independent experiments. *****p* < 0.0001.

**Fig. S5 Effect of SMOC2 knockdown on the expression of FTO, IGF2BP2 and METTL14 in RA FLSs.** (A-C) RT-qPCR was performed to validate the mRNA expression of FTO (A), IGF2BP2(B), and METTL14 (C) after SMOC2 knockdown in RA FLSs. Data are presented as the mean ± SD of 6 independent experiments involving 6 different patients with RA. ****P* < 0.001, **P* < 0.05.

**Fig. S6 Effect of SMOC2 knockdown on the expression of MYO1C, ALKBH5, efficiency of ALKBH5, YTHDF2 and YTHDF3 knockdown and the binding ability of YTHDF2 and YTHDF3 to MYO1C.** (A-B) Effect of SMOC2 knockdown on the protein expression of ALKBH5 (A), MYO1C (B). Data shown are the mean ± SD of densitometry quantification (right panel) from 3 independent experiments involving 3 different patients with RA. (C-E) RA FLSs were transfected with ALKBH5 siRNA, YTHDF2 siRNA and YTHDF3 siRNA for 48 hours and then the mRNA expression of ALKBH5 (C), YTHDF2 (D) and YTHDF3(E) was measured by RT-qPCR analysis. (F-G) The correlation of YTHDF2 (F) and YTHDF3 (G) mRNA expression with MYO1C mRNA expression in RA FLSs. YTHDF2, YTHDF3 and MYO1C mRNA expression in RA FLSs were measured by RT-qPCR analysis, respectively. Correlation analysis was performed by Spearman’s rank order correlation test. (H) The FIMO software in meme-suite was used to identify the potential m6A reader binding site of YTHDF2 in the region of m6A peaks in MYO1C. Data shown are the mean ± SD from at least 3 independent experiments. ****p < 0.0001, ***P < 0.001, **P < 0.01, *P < 0.05.

**Fig. S7** **Effect of SMOC2 knockdown on the expression of ALKBH5 and SOX4 in synovial tissue from CIA rats.** (A) Expression of SMOC2 and ALKBH5 was measured by immunofluorescence staining in synovial tissue from CIA rats. The representative images and quantification of the percentage of SMOC2-positive (red) and ALKBH5-positive (green) cells. Original magnification, ×100. (B) Expression of SMOC2 and SOX4 was measured by immunofluorescence staining in synovial tissue from CIA rats. The representative images and quantification of the percentage of SMOC2-positive (red) and SOX4-positive (green) cells. Original magnification, ×100.
