# Supplementary figures and images for: SMOC2 promotes aggressive behavior of fibroblast-like synoviocytes in rheumatoid arthritis through transcriptional and post-transcriptional regulating MYO1C
Source: Cell Death Dis. 2022 Dec 13;13(12):1035. doi: 10.1038/s41419-022-05479-0 (PMC9747908; doi:10.1038/s41419-022-05479-0)

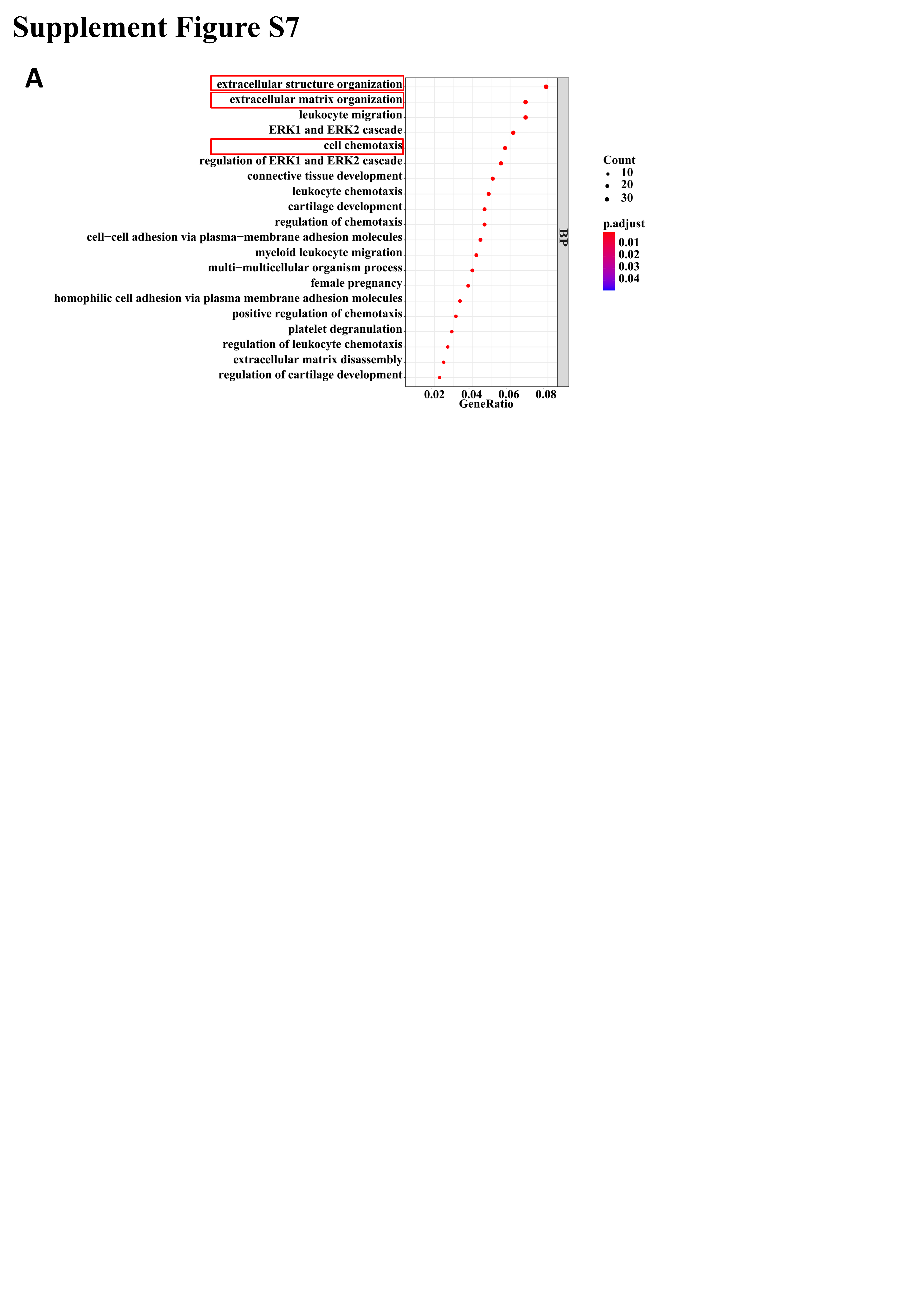

Supplement: Supplementary file 2 — Supplement Figure S1 [file 41419_2022_5479_MOESM2_ESM.tif]

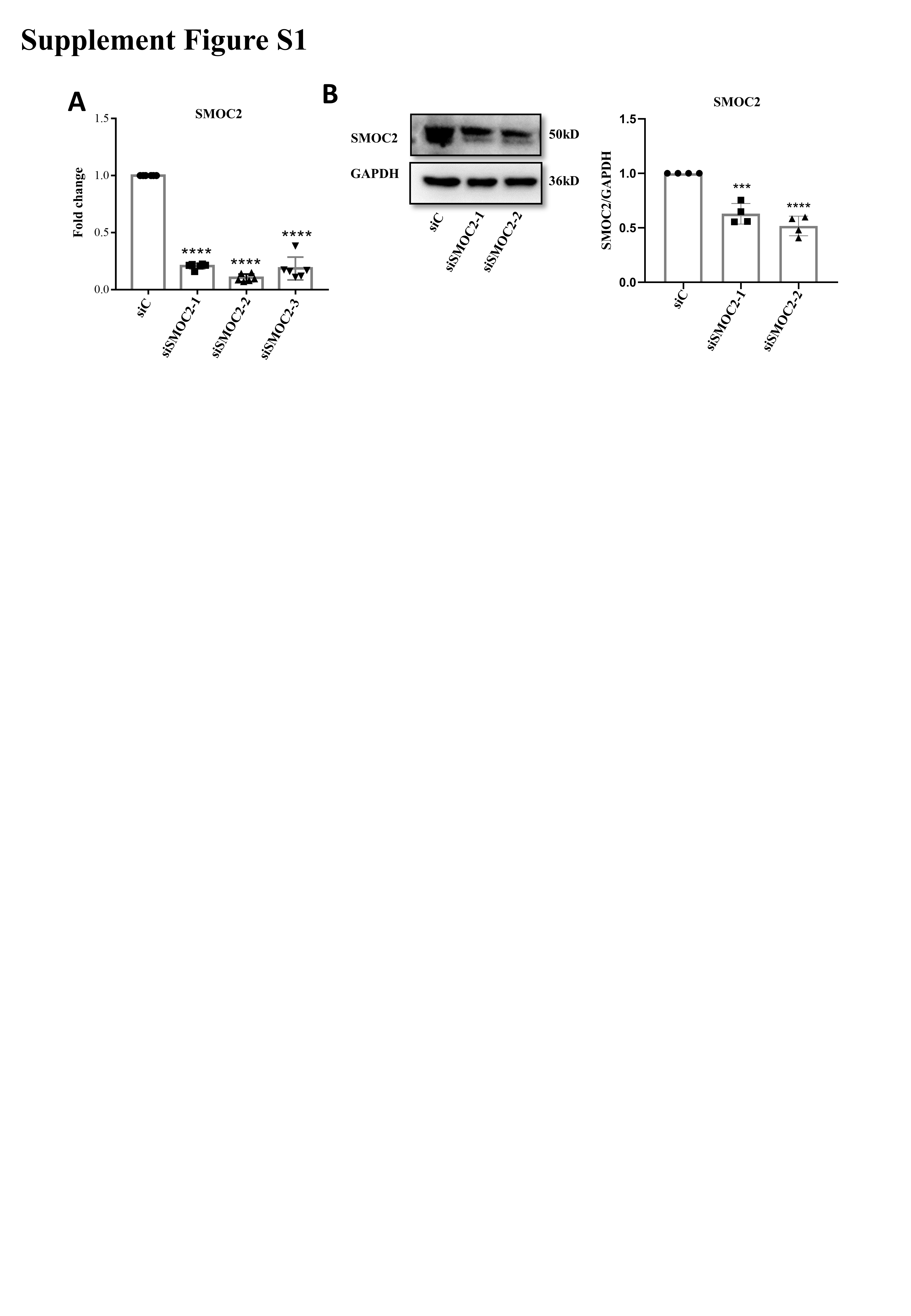

Supplement: Supplementary file 3 — Supplement Figure S2 [file 41419_2022_5479_MOESM3_ESM.tif]

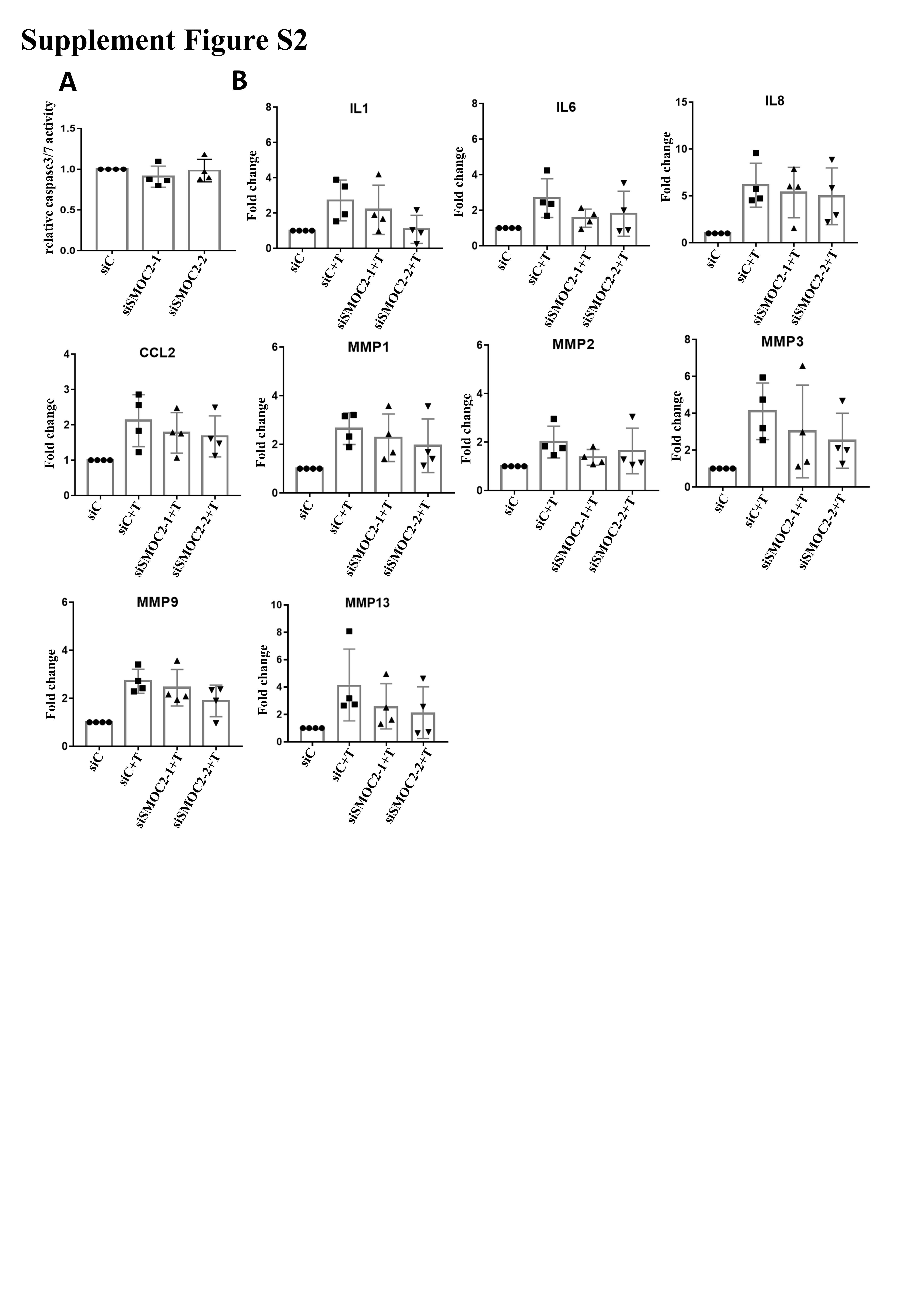

Supplement: Supplementary file 4 — Supplement Figure S3 [file 41419_2022_5479_MOESM4_ESM.tif]

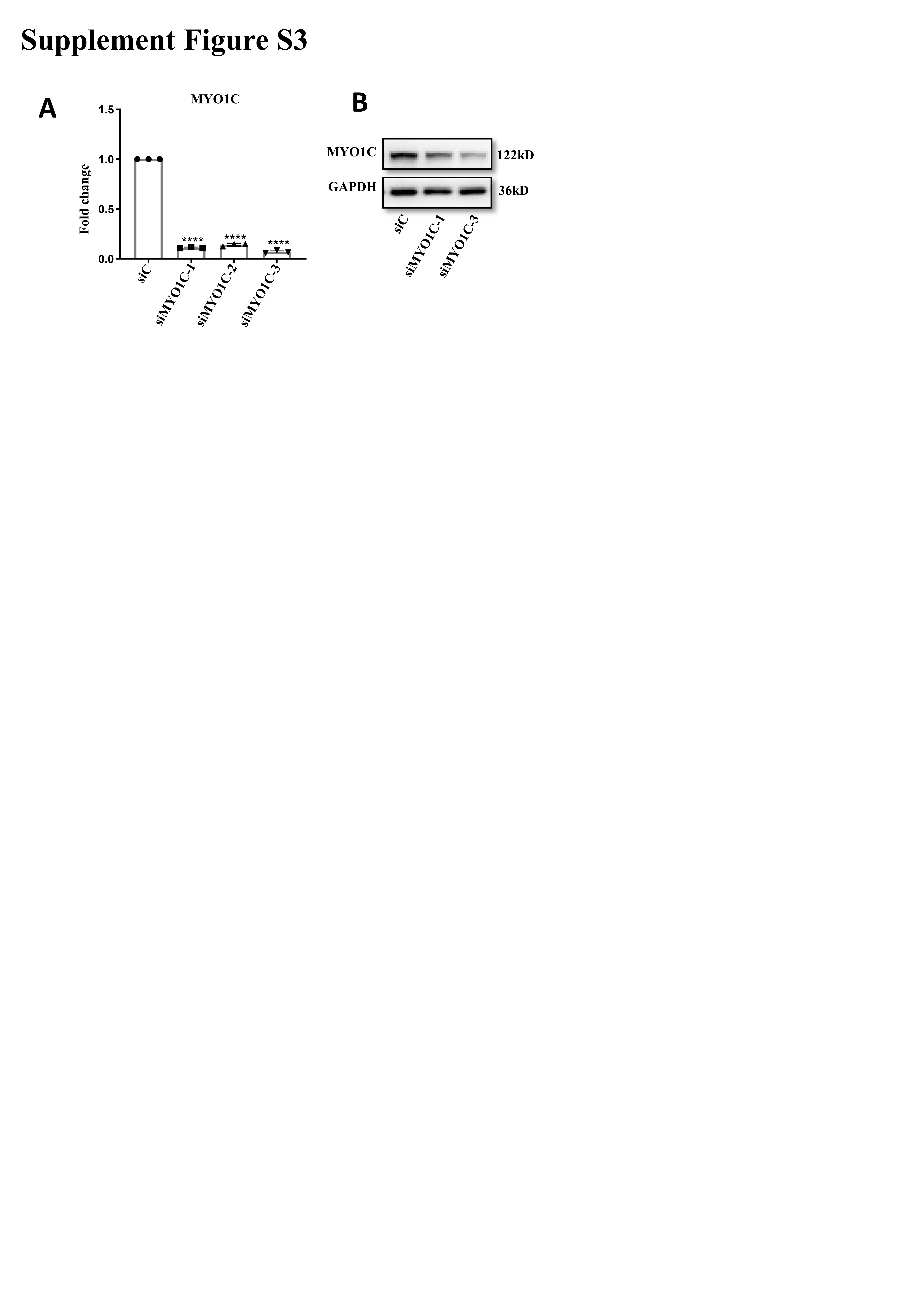

Supplement: Supplementary file 5 — Supplement Figure S4 [file 41419_2022_5479_MOESM5_ESM.tif]

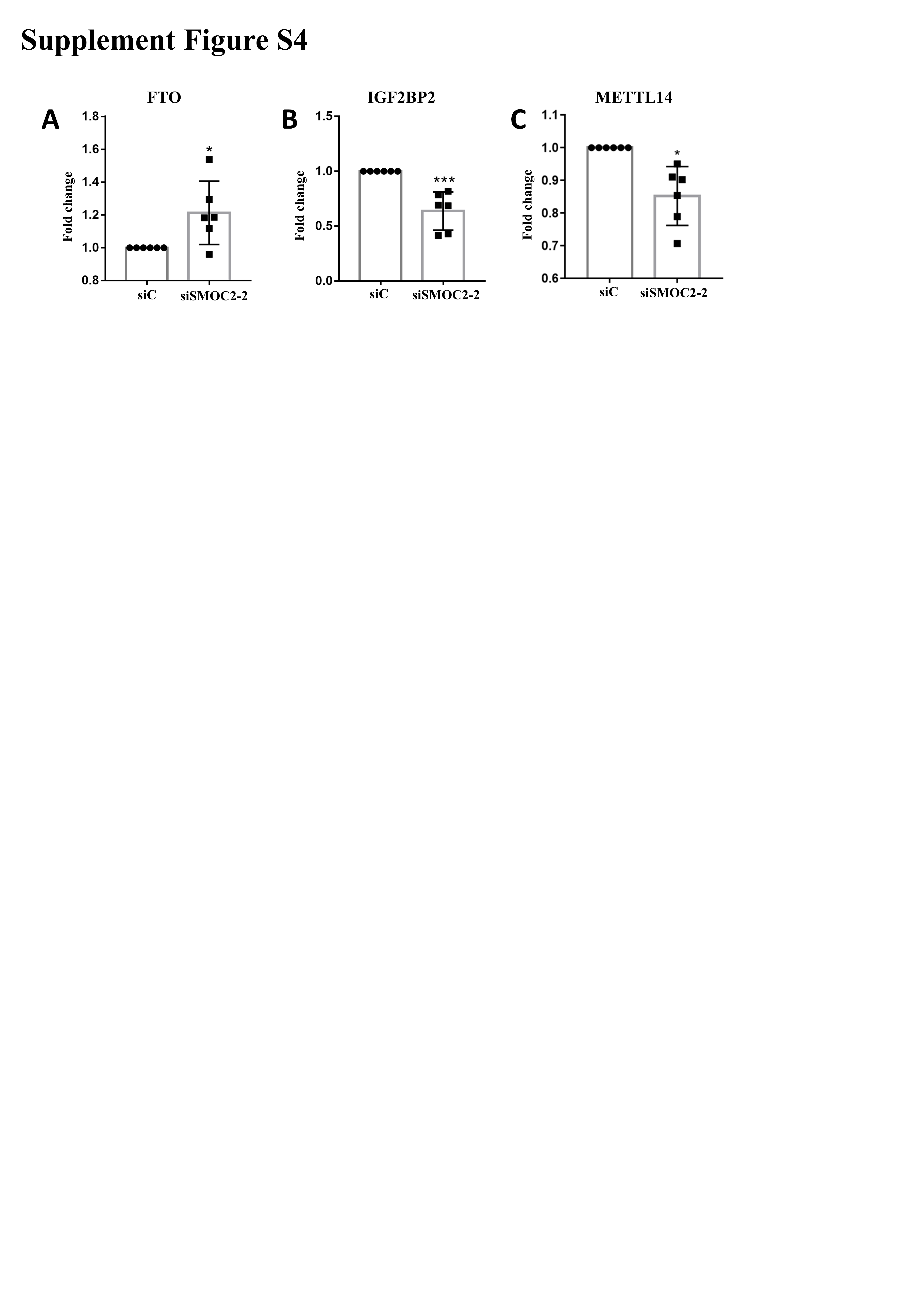

Supplement: Supplementary file 6 — Supplement Figure S5 [file 41419_2022_5479_MOESM6_ESM.tif]

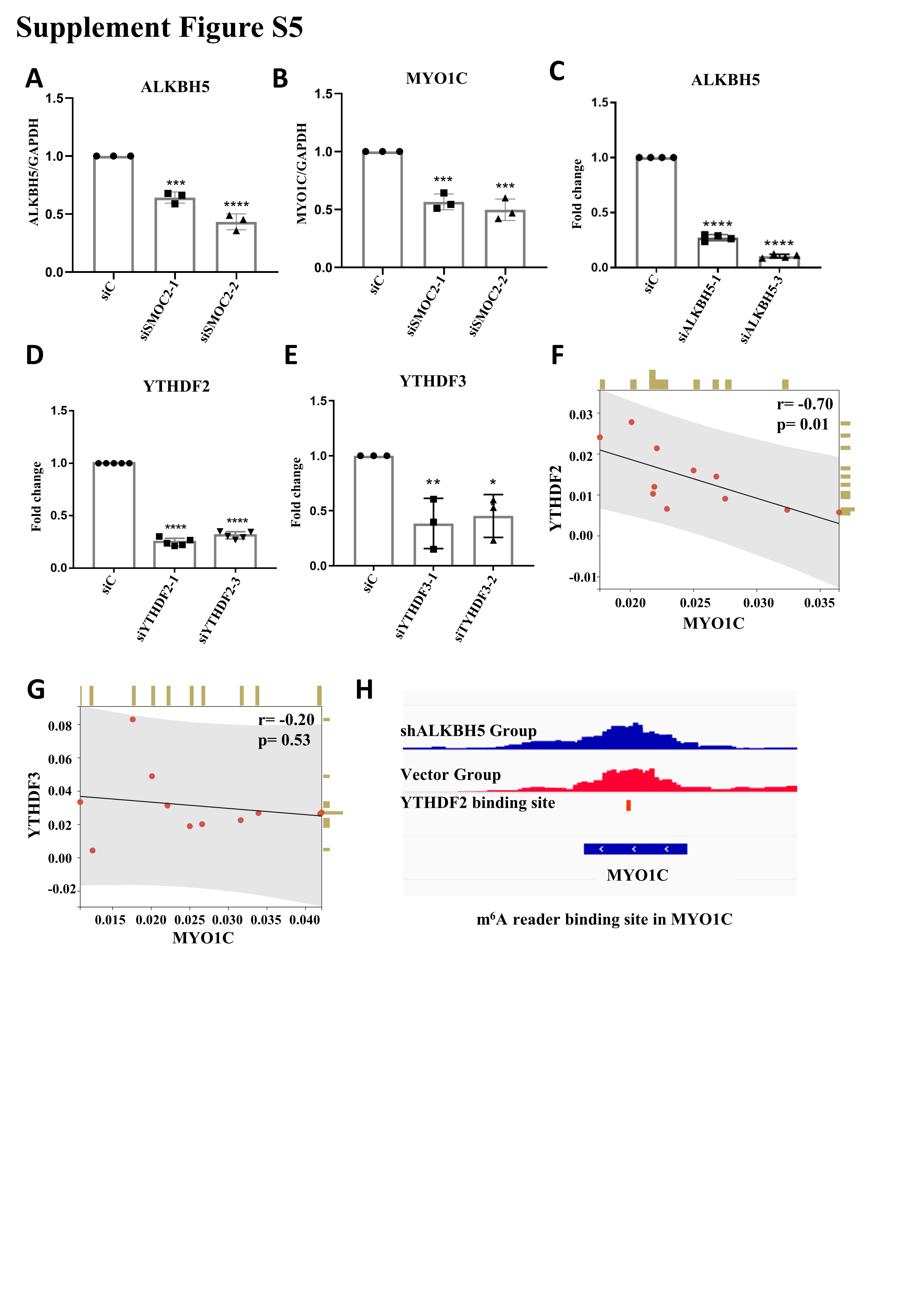

Supplement: Supplementary file 7 — Supplement Figure S6 [file 41419_2022_5479_MOESM7_ESM.tif]

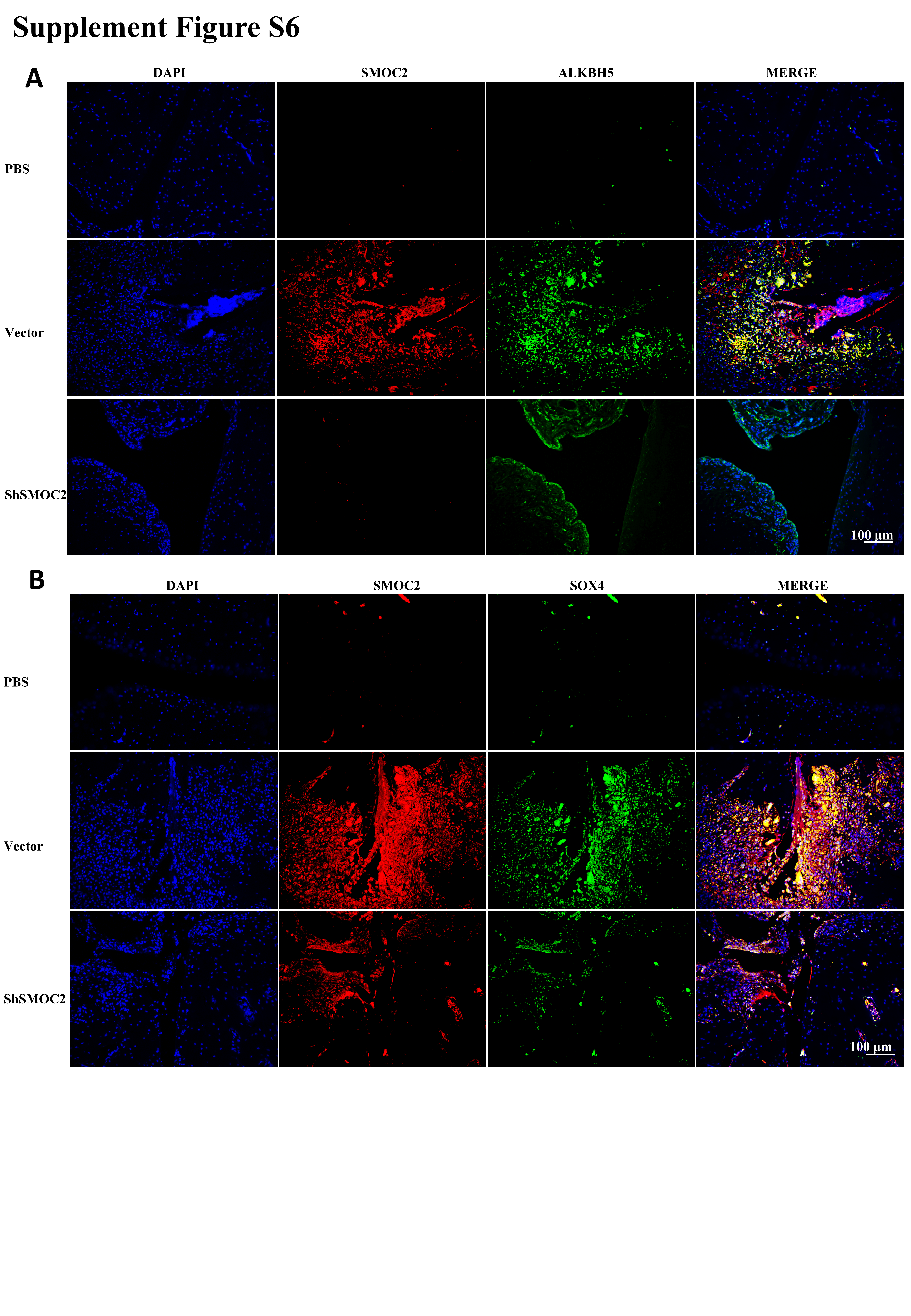

Supplement: Supplementary file 8 — Supplement Figure S7 [file 41419_2022_5479_MOESM8_ESM.tif]

### Figure 1E

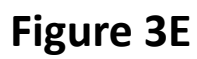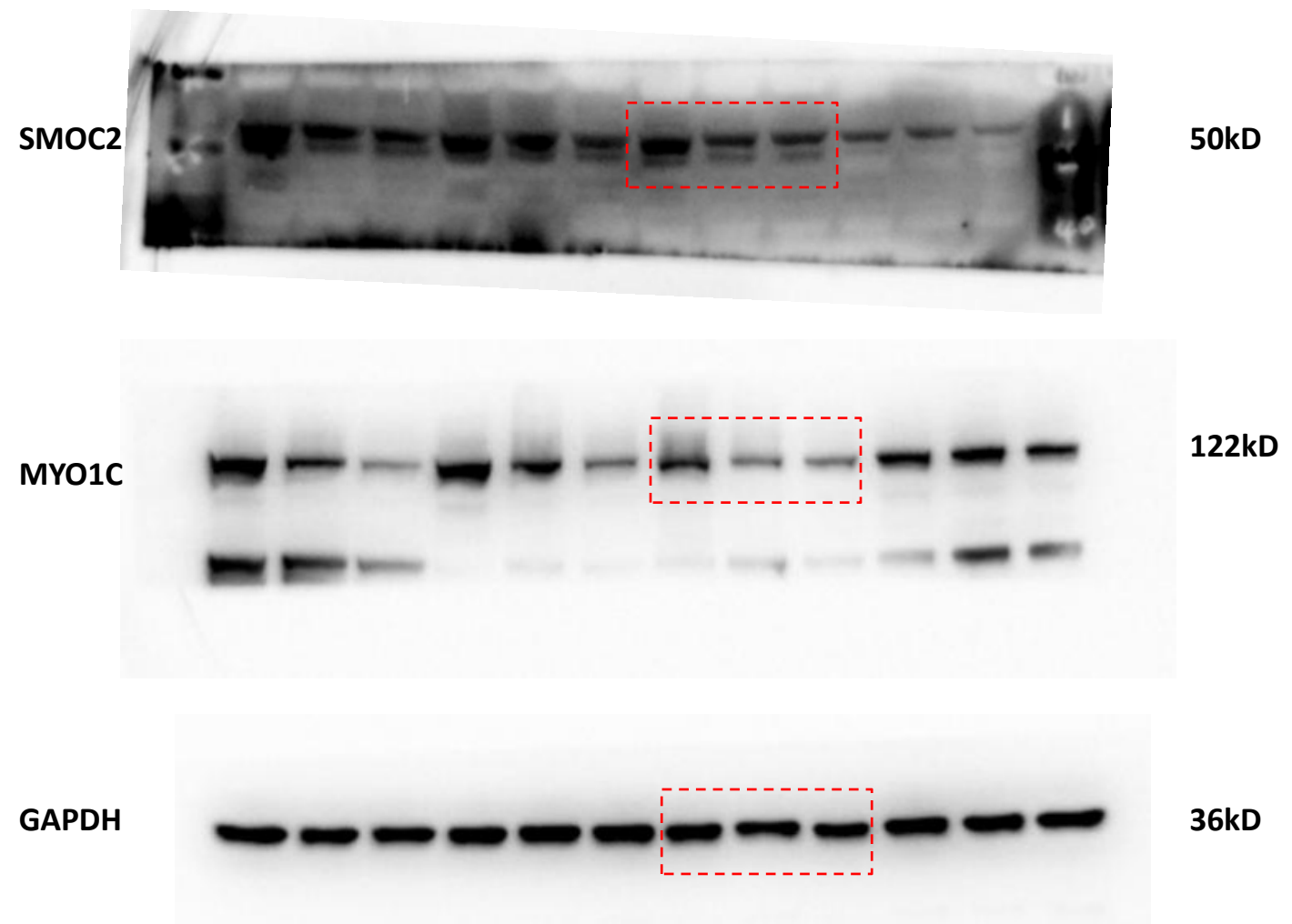

**Figure 4B**

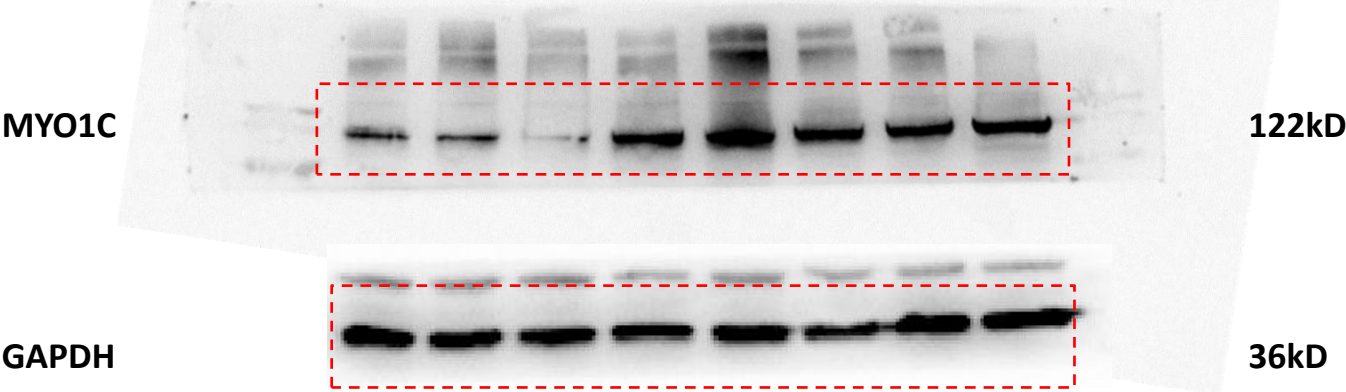

**Figure 5G**

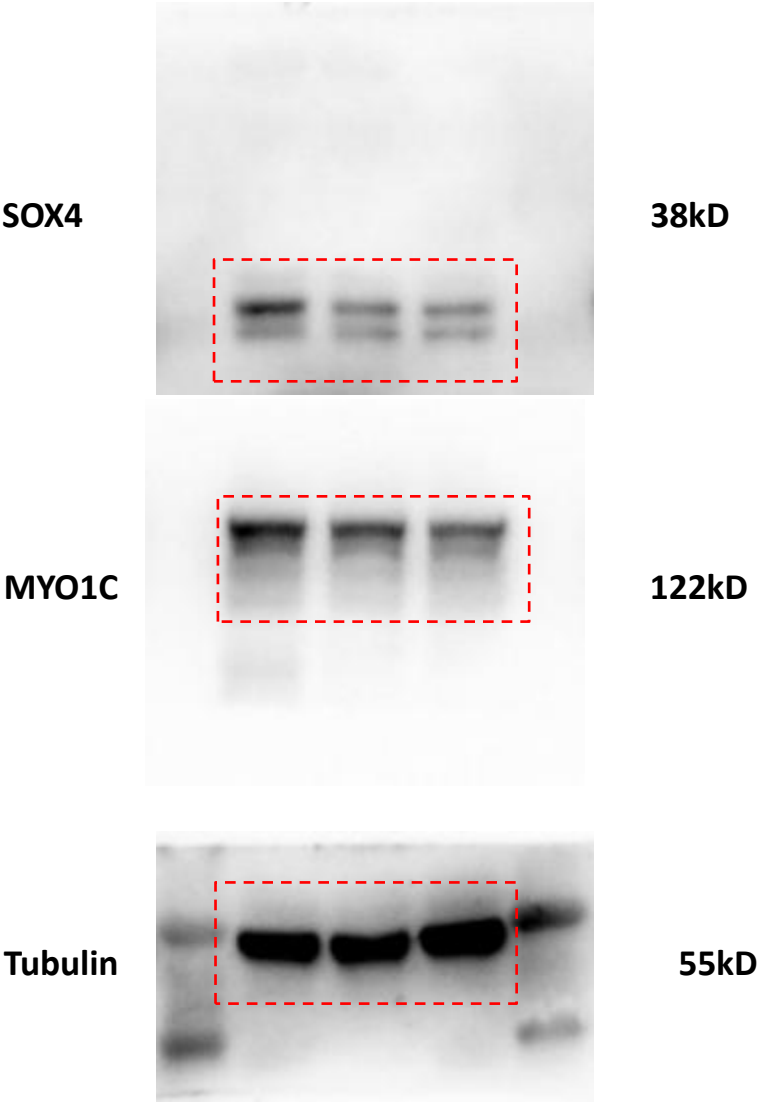

**Figure 6E**

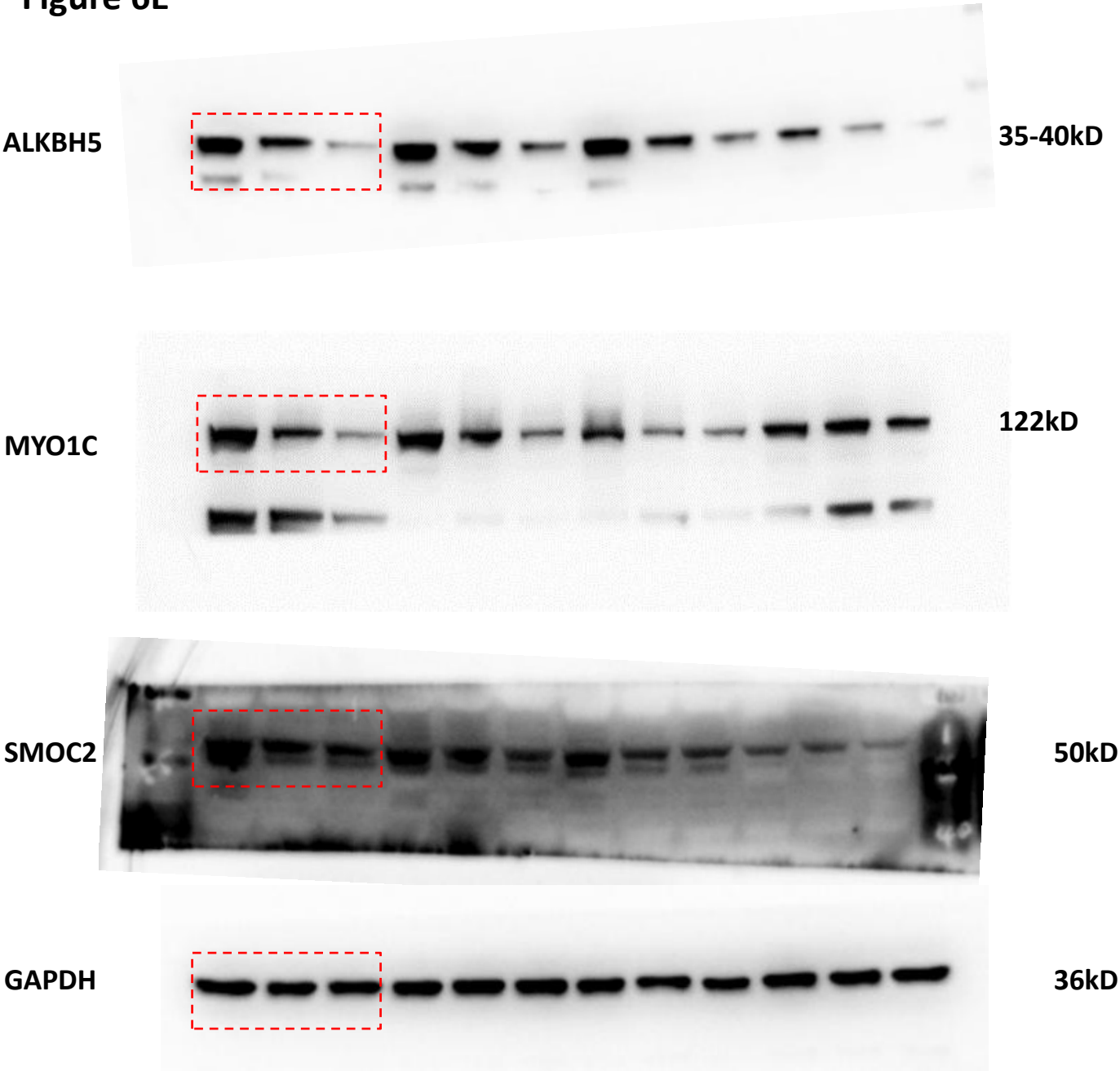

**Figure 6G**

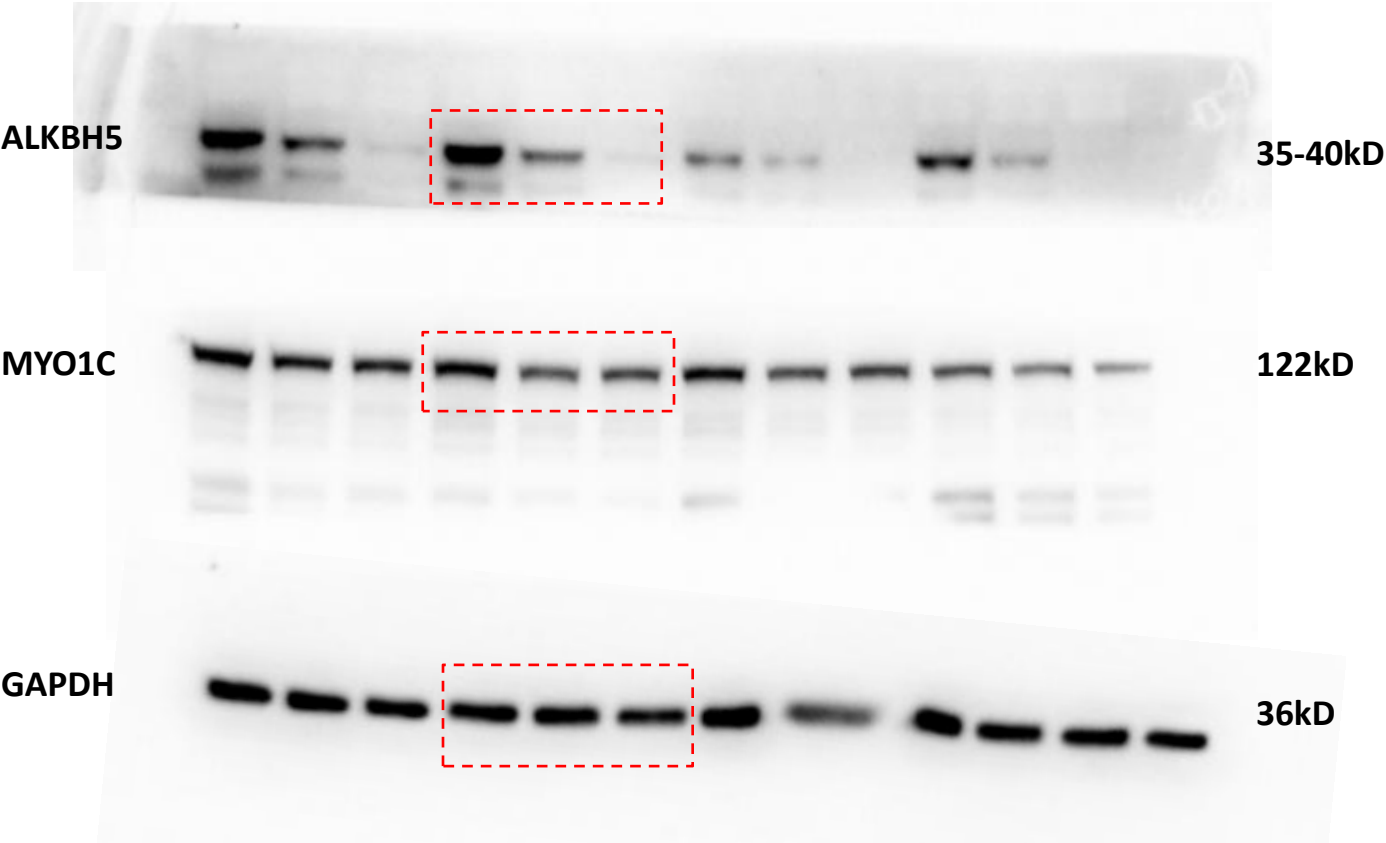

**Figure 6M**

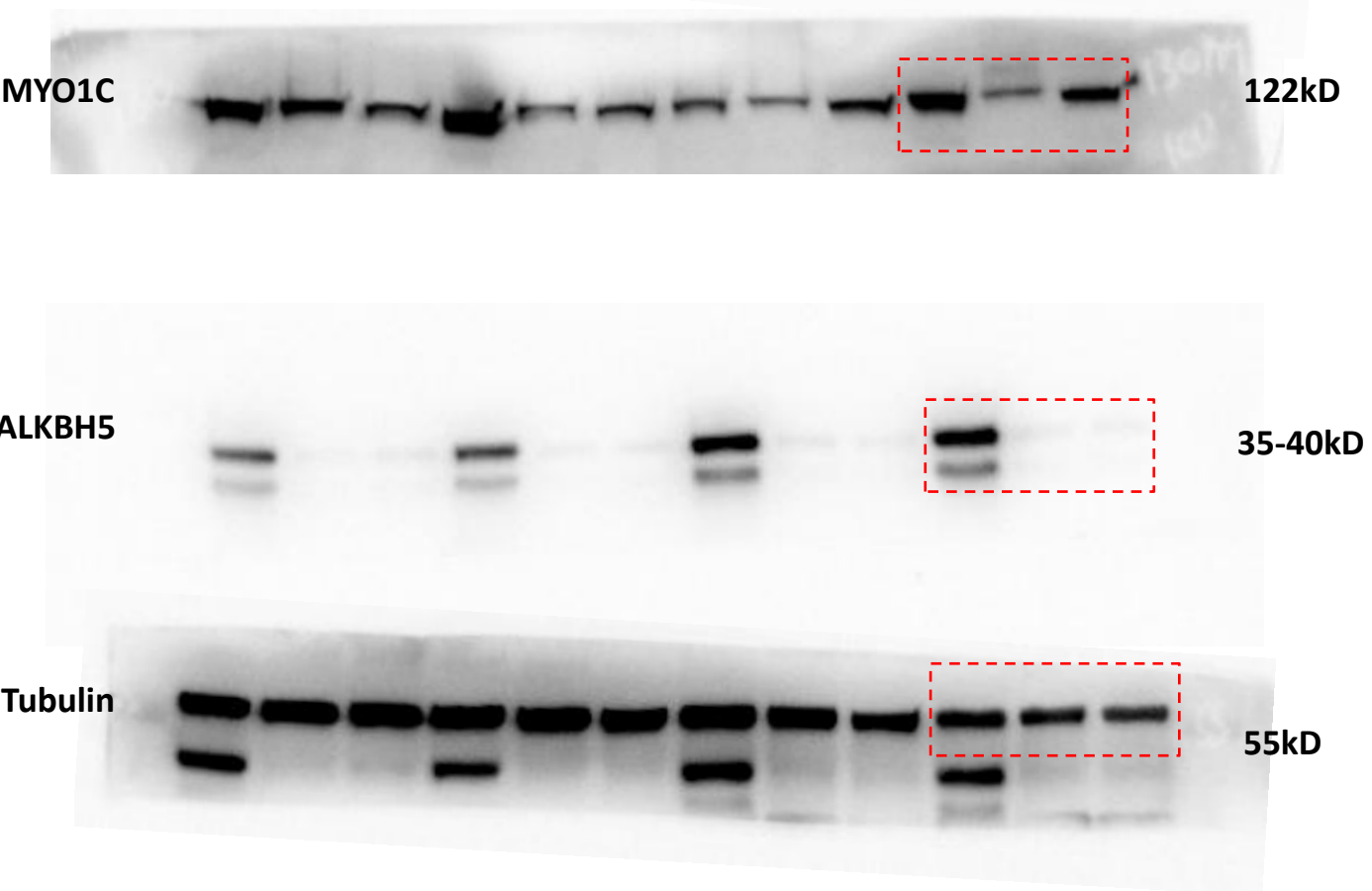

**Fig. S1B**

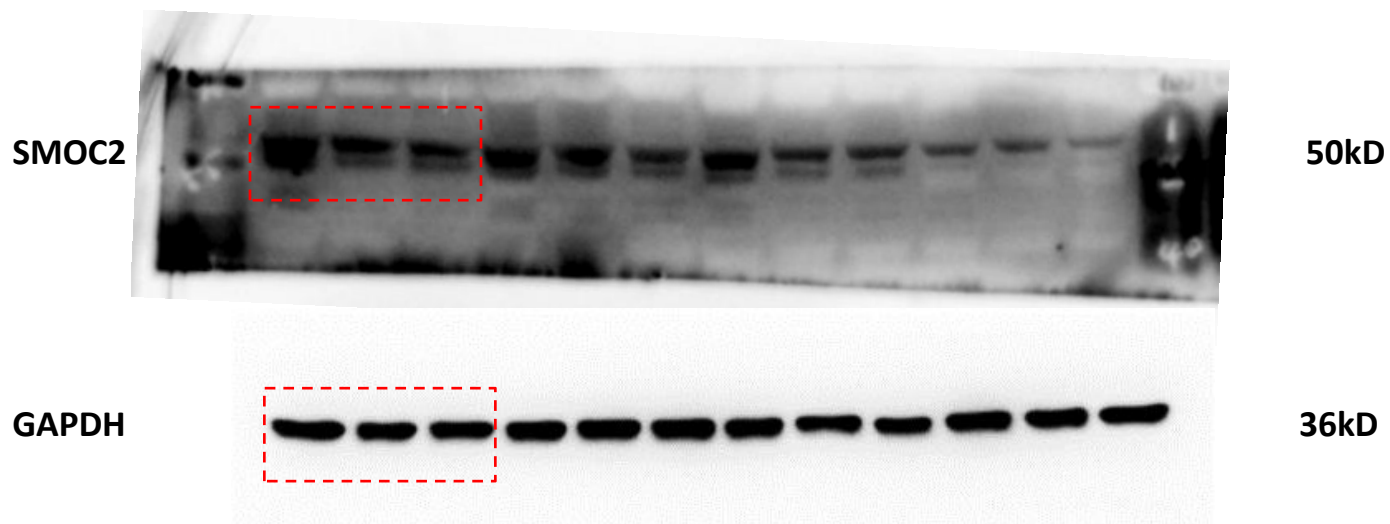

**Fig. S3B**

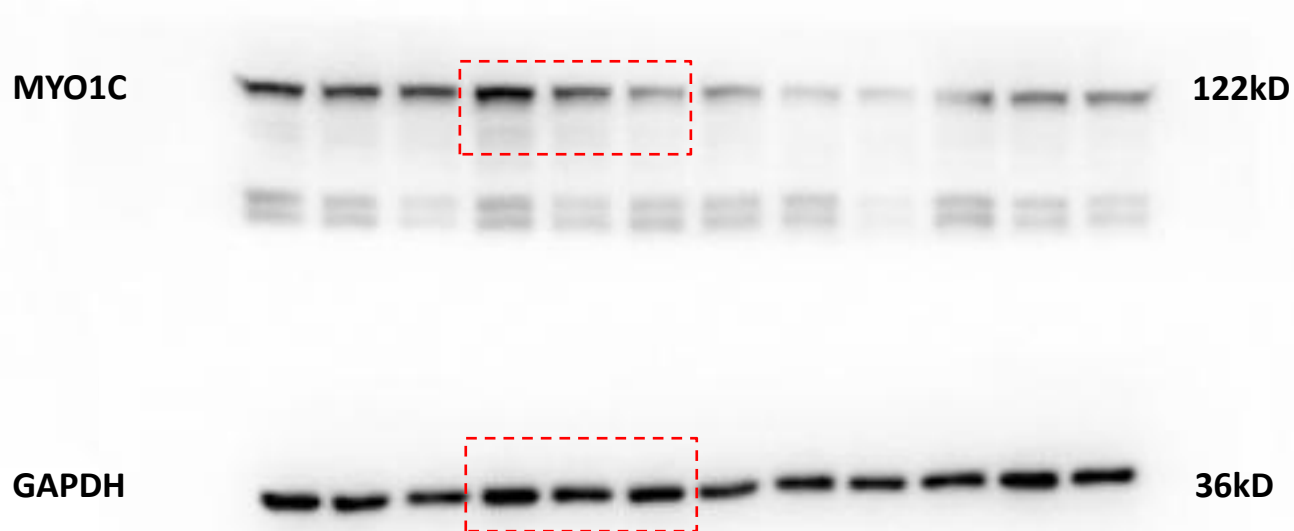

Supplement: Supplementary file 10 — Original Data File [file 41419_2022_5479_MOESM10_ESM.pdf]
